# COVID-19 and the Importance of Being Prepared: A Multidisciplinary Strategy for the Discovery of Antivirals to Combat Pandemics

**DOI:** 10.3390/biomedicines10061342

**Published:** 2022-06-07

**Authors:** Maria Galvez-Llompart, Riccardo Zanni, Jorge Galvez, Subhash C. Basak, Sagar M. Goyal

**Affiliations:** 1 Molecular Topology & Drug Design Research Unit, Department of Physical Chemistry, University of Valencia, 46100 Burjasot, Spain; riccardo.zanni@uv.es (R.Z.); jorge.galvez@uv.es (J.G.); 2 Department of Chemistry and Biochemistry, University of Minnesota, Duluth, MN 55812, USA; sbasak@nrri.umn.edu; 3 Veterinary Population Medicine Department, College of Veterinary Medicine, University of Minnesota, Saint Paul, MN 55108, USA; goyal001@umn.edu

**Keywords:** QSAR, drug discovery, antiviral, SARS-CoV-2, COVID-19, viral protease, molecular docking, protease inhibitors, human coronavirus 229E

## Abstract

During an emergency, such as a pandemic in which time and resources are extremely scarce, it is important to find effective and rapid solutions when searching for possible treatments. One possibility in this regard is the repurposing of available “on the market” drugs. This is a proof of the concept study showing the potential of a collaboration between two research groups, engaged in computer-aided drug design and control of viral infections, for the development of early strategies to combat future pandemics. We describe a QSAR (quantitative structure activity relationship) based repurposing study on molecular topology and molecular docking for identifying inhibitors of the main protease (Mpro) of SARS-CoV-2, the causative agent of COVID-19. The aim of this computational strategy was to create an agile, rapid, and efficient way to enable the selection of molecules capable of inhibiting SARS-CoV-2 protease. Molecules selected through in silico method were tested in vitro using human coronavirus 229E as a surrogate for SARS-CoV-2. Three strategies were used to screen the antiviral activity of these molecules against human coronavirus 229E in cell cultures, e.g., pre-treatment, co-treatment, and post-treatment. We found >99% of virus inhibition during pre-treatment and co-treatment and 90–99% inhibition when the molecules were applied post-treatment (after infection with the virus). From all tested compounds, Molport-046-067-769 and Molport-046-568-802 are here reported for the first time as potential anti-SARS-CoV-2 compounds.

## 1. Introduction

The pandemic of COVID-19 due to SARS-CoV-2 has made clear the importance of being prepared. At the beginning of the pandemic, the virus spread very easily among the global human population because no preventive strategy or treatment was available. Scientists from all over the world started a race against the clock to understand this new pathogen without a clear plan or path to follow. The virus was killing millions of people [1,2,3,4] and the researchers were struggling to find a solution to the problem. Nevertheless, because humans are among the most resilient and resourceful beings, we did learn a lot. In less than a year, vaccines were developed against SARS-CoV-2 [5]. In this scenario, computational drug design and discovery, especially the in silico repositioning strategy, played a crucial role in accelerating the process of identifying potential treatments against this virus [6,7]. During an emergency, such as a pandemic, in which time and resources are extremely scarce, it is important to re-adapt (re-purpose) the drugs that are already on the market [7]. Thus, studies were published in which many FDA-approved, drugs were screened as a potential treatment for COVID-19. This, in turn, helped spur pre-clinical and clinical research in which energy was focused on specific targets and compounds [8,9,10,11,12,13,14,15,16].

In this study, we show the potential of a new, multidisciplinary collaboration between two research groups: one group engaged in computer-aided drug design led by Prof. Jorge Galvez [17,18,19] and Prof. Subhash Basak [20,21,22], and the other led by a virologist Prof. Sagar Goyal [23] with expertise in pathogenesis and control of viral infections. From the synergy of these groups, a research unit for the development of early strategies effective against future pandemics has been established. As a proof of concept, the results of a QSAR (quantitative structure activity relationship) repositioning study based on molecular topology and molecular docking of drugs against the main protease (Mpro) of SARS-CoV-2 are presented.

Two viral proteases, Mpro and PLpro [24,25,26], are responsible for the cleavage of SARS-CoV-2 polyprotein yielding a complement of structural and accessory proteins that are essential for virus replication. The crystal structure of the main protease of SARS-CoV-2 in a complex with inhibitor N3 [27] was retrieved from the Protein Databank website [28]. A virtual screening based on molecular docking for all the molecules of the DrugBank database [29] was performed using the Maestro software from the Schrödinger suite [30]. We selected 80 molecules showing the most favorable docking interaction values (lower than −8 Kcal/mol) and 126 with the least favorable interaction values (greater than −2 Kcal/mol). This combined pool of compounds was used to create a new data set for the development of a QSARs based on topological descriptors using linear discriminant analysis (LDA) and artificial neural networks (ANN).

Molecular topology is a mathematical paradigm that uses graph theory to translate a chemical structure into a set of numbers by means of matrices [31,32,33,34]. These numbers, called topological descriptors, encode chemical and topological information that can be related to a specific biological or pharmacological property of the molecule [34,35,36,37,38]. The development and application of the discriminant QSAR models allow qualitative discrimination between compounds that have the potential to act as a viral protease inhibitor and those that do not. We then developed a second molecular topology QSAR strategy based on multi-linear regression analysis and regression neural networks to quantitatively predict the docking score value for each potential drug against SARS-CoV-2 protease. The best candidates were evaluated in vitro using human coronavirus 229E as a surrogate for SARS-CoV-2.

The aim of this computational strategy is to create an agile, rapid, and efficient method for the selection of molecules with potential antiviral activity by predicting specific docking scores and the potential interaction of a drug with a certain target (in this case the SARS-CoV-2 main protease) without conducting full molecular docking simulation. This method converts the information on structure-based drug design (docking score from molecular docking study) to a ligand-based drug design (QSAR modeling). Modeling the docking score of the main protease (Mpro) of SARS-CoV-2 provides a rapid method to screen large databases. A final objective of this study was to show the potential power of a multidisciplinary collaboration as a rapid and reliable system to identify potential antiviral treatments during future pandemics.

## 2. Materials and Methods

### 2.1. QSAR Strategy

**Figure 1:**
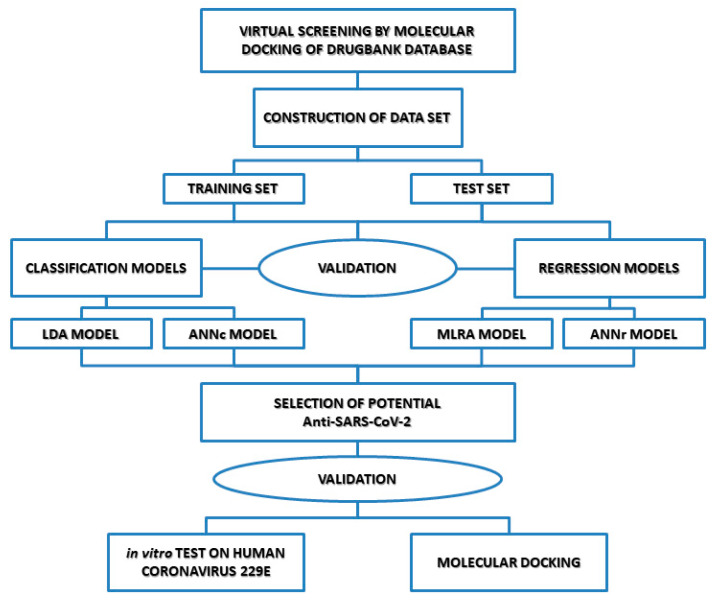
Search algorithm used to develop in silico strategy for the repositioning of potential Mpro inhibitors against SARS-CoV-2.

Figure 1 shows the algorithm strategy used for the identification of potential inhibitors of SARS-CoV-2 main protease.

### 2.2. Creation of a Data Set of Anti-Protease Compounds: Molecular Docking Studio

As suggested in previous sections, the dataset used for the construction of QSAR models was obtained from a previous molecular docking studio on the DrugBank database; this library contains 17,271 compounds of approved, investigational, and experimental (discovery-phase) drugs) [29]. The DrugBank database was screened to determine the binding affinity of the compounds with the crystallized protein structure of SARS-CoV-2 main protease (Mpro) by means of docking score values. To be exact, the crystal structure of the main protease of the virus in complex with inhibitor N3 (6LU7), was retrieved from the Protein Databank website [27].

### 2.3. Molecular Docking Simulation

**Figure 2:**
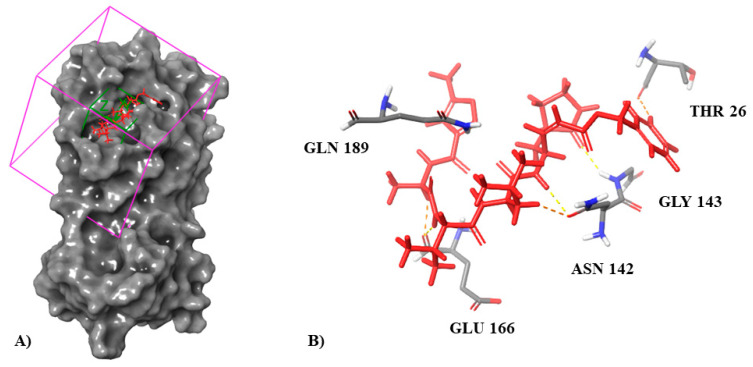
(**A**) Binding pocket for Mpro crystallized protein (PDB:6LU7) docking studio. (**B**) Interaction of Mpro co-crystallized ligand (inhibitor N3) with key catalytic residues.

The molecular docking simulation was performed by using the Schrodinger Suite 2021-3 software (Maestro suite) Schrödinger, LLC, New York, United States of America [30]; the default parameters were adopted unless otherwise reported [39]. For docking studies, the 6LU7 crystal structure of SARS-CoV-2 main protease in PDB was used. The main protease structure was subjected to Protein Preparation Wizard in Maestro. During this process, the missing hydrogens were added, partial charges were assigned using OPLS-3e force field, and both hydrogens and heavy atoms were optimized by restrained minimization. 2D structures of the training and test set compounds from the DrugBank database were converted to 3D structures via the Ligprep module in Maestro. Ligprep corrects the protonation and ionization states of the compounds, assigns proper bond orders, and creates the tautomeric and ionization states for each ligand. The receptor grid generation was calculated based on centroid of co-crystallized ligand (peptide inhibitor N3) with Mpro (6LU7), defined by the grid-box coordinates [x = −10,712, y = 12,411, z = 68,831], grid-box size of 30.98 Å, and ligand grid of 10 Å (Figure 2). Rigid receptor docking protocol was run in standard precision (SP) mode of Glide based on OPLS-3e force field, while ligands were flexible.

The resulting compounds with docking score values between −8 and −12 kcal/mol were labeled and placed into the active group to be used in the QSAR study, while compounds with a docking score value of >−2 were placed in the inactive group. Docking studio information made it possible to create a database of potential protease inhibitors. A total of 274 compounds (206 for the training set and 68 for the external test) were used. All of the data sets are shown in the Supplementary Material section.

### 2.4. Topological Descriptors and Statistical Modeling Methods

Topological and topo-chemical indices for the dataset were calculated using alvaDesc software version 2.0.6, Lecco (Italy), 2021 [40]. Default variable reduction tool from alvaDesc software has been applied (1821 descriptors have been employed). Information related to training and external test set data in addition to selected compounds information is reported in the Supplementary Material section. To develop predictive models, various statistical methods were used as discussed below.

#### 2.4.1. Linear Discriminant Analysis (LDA)

Linear algorithms such as linear discriminant analysis (LDA) allow a linear combination of features capable of separating two or more classes of objects in specific classification categories. In this study, LDA was employed to generate the discriminant model DF_Class_6LU7_, which discriminates between molecules capable and incapable of interacting with the main protease of the virus. An important aspect of building a robust LDA model is the selection of the most significant variables or descriptors to characterize the compounds so that their contribution to the discrimination is high. To select the best descriptors, we followed a forward stepwise algorithm based on *p*-value. Therefore, at each step, the variable with a more favorable *p*-value < 0.05 was chosen. The process ends when the algorithm cannot introduce any more descriptors with a *p*-value < 0.05. Therefore, at each step, the variable that adds the most to the separation of the groups is entered into the discriminant function. Another parameter to assess the significance of the selected descriptor is the Fisher-Snedecor parameter; higher values indicate a better descriptor. The quality of the discriminant function is assessed by the Wilks’ lambda parameter [41]. In general, the Wilks’ lambda can take values between 0 and 1; the smaller the value the better the prediction. Statistica was the software used for developing linear discriminant models [42].

#### 2.4.2. Multilinear Regression Analysis (MLRA)

A multilinear regression analysis model was developed MLR_reg_6LU7_ [43] using a forward stepwise variable selection procedure in which variables are sequentially entered into the model depending on the *p*-value selected (threshold: *p* < 0.05). Subsequently, the best subseries of six descriptors with respect to the property (6LU7-docking score) are identified. Therefore, from almost 2000 descriptors calculated, only six top descriptors were selected for modeling docking score values against Mpro (6LU7). Statistical parameters indicating the quality of the regression are, among others, correlation coefficient, r^2^, the F (Fisher-Snedecor), and *p*-values.

#### 2.4.3. Artificial Neural Network Analysis (ANN): Classification and Regression

The ANN is a computer-based model in which a number of nodes (also called processing elements, units, or neurons) are interconnected by links in a netlike structure forming “layers”. A variable value is assigned to each node [44,45]. The nodes can be of three different kinds: (1) Input nodes, which form the input layer, receive their values by direct assignation, and are associated with independent variables, with the exception of the bias node; (2) The hidden nodes (constituting hidden layers) collect values from other nodes and pass it on to a non-input node; and (3) Output nodes, which collect values from other units and correspond to different dependent variables, forming the output layer (regression ANN) or layers (classification ANN). The links between the units have values associated, named weights, that condition the values assigned to the nodes. Additional weights are assigned to the bias values, which act as node value offsets. The weights are adjusted through a training process.

### 2.5. QSAR Models and Validation

#### 2.5.1. Classification Matrix and External Validation

The discriminant reliability of LDA and ANN_Class_ models is evaluated by the classification matrices, which sort all cases from the model into categories by determining whether the predicted value matches the actual value. 

Validation is required after the construction and analysis of the models. In this respect, the external validation procedure is going to depict how our models classify or predict information about hitherto never seen compounds. Of 274 compounds, 206 were divided into training sets and 68 into external sets. Therefore, 25% of the data set was randomly left out of the model construction as a test set. All data sets are reported in the Supplementary Material section (Tables S1–S8).

#### 2.5.2. Relative Operating Characteristic Curve (ROC) Curve

To assess the predictive capability of the LDA models and to determine their sensitivity and specificity, the relative operating characteristic curve (ROC curve) is calculated [46]. The sensitivity is intended as the true positive rate and is defined as the percent of active molecules correctly classified by the model. Specificity, or true negative rate, is the percent of non-active molecules correctly classified by the model. In the ROC curve, the y-axis represents the sensitivity of the model as the discrimination threshold is varied, which simultaneously affects the specificity of the model. For convenience, the x-axis represents 1-specificity, so that both magnitudes change in the same direction as the discrimination threshold is varied. In this context, the area under the ROC curve (AUC) is often regarded as an indicator of the performance of the classifier. A value of AUC = 1 would be obtained for a perfect classifier, whereas the diagonal line would represent a model with no classification power in predicting binary outcomes. The best possible prediction method would yield a point in the upper left corner or coordinate (0, 1) of the ROC space, representing 100% sensitivity (no false negatives) and 100% specificity (no false positives). The (0, 1) point is also called a perfect classification. A random guess would give a point along a diagonal line (the so-called line of no-discrimination) from the bottom left to the top right corners (regardless of the positive and negative base rates).

### 2.6. Pharmacological Distribution Diagrams

Linear discriminant analysis in topological QSAR enables the plotting of frequency distribution diagrams, called Pharmaceutical Distribution Diagrams. These diagrams represent the frequency of the number of molecules within an interval of values of the discriminant function vs. these values [47]. The plot provides a straightforward way of visualizing the regions of minimum overlap between active and inactive compounds; DF regions with the highest expectancy of finding active molecules and range of applicability domain for a DF. For an arbitrary range of values of a given function, an “expectancy of activity” can be defined as E_a_ = a/(i + 1), where “a” is the number of active compounds in the range divided by the total number of active compounds and “i” is the number of inactive compounds in the interval divided by the total number of inactive compounds. The expectancy of inactivity is defined in a symmetrical way, as E_i_ = i/(a + 1).

### 2.7. Virtual Screening

After the QSAR models were established, virtual screening of the Emolecule database was carried out to identify potential repurposed drugs and novel synthetic chemicals against SARS-CoV-2. A stepwise strategy was adopted for performing a virtual screening using each of the models described.

### 2.8. In Vitro Testing

Human respiratory coronavirus 229E was used as a surrogate of SARS-CoV-2. The virus was propagated and titrated in MRC-5 cells (human fetal lung fibroblast cells). The titers, expressed as Log_10_ TCID_50,_ were calculated by the Karber [48] method. The MRC-5 cells were grown in Eagle’s MEM containing fetal bovine serum and antibiotics. We used three different in vitro methods to determine the efficacy of the six potential antiviral compounds (see below). All these dilutions were inoculated in three wells each and all experiments were done in duplicate. Both negative controls which consisted of cell cultures not infected with the virus, and positive controls, which consisted of cell cultures infected with the virus but not treated with any drug, have been considered in the experiments.

a. Pre-treatment of cells: After discarding its growth medium, the cell monolayers were covered with 100 µL of a solution containing 50 µg/mL of a given compound). Each compound was tested in separate cell monolayers. After incubation at room temperature for 60 min, the monolayers were washed with sterile PBS. Immediately, the washed monolayers were inoculated with serial 10-fold dilutions of 229E (prepared in MEM). The plates were then incubated at 37 °C in a 5% CO_2_ incubator and examined daily under an inverted microscope for the appearance of virus-induced cytopathic effects (CPE). After 5 days of incubation, 229E titers in pre-treated and untreated monolayers were calculated and compared.
b. Co-treatment of cells with antivirals and virus: Serial 10-fold dilutions of 229E were prepared separately in 1:10 dilutions of each antiviral. From each virus dilution, 100 µL was used to infect MRC-5 monolayers. The titers of 229E prepared in antiviral solutions and 229E prepared in MEM (control) were calculated and compared after 5 days of incubation at 37 °C in a 5% CO_2_ incubator.
c. Post-infection treatment of cells with antivirals: Cell monolayers were inoculated with serial 10-fold dilutions of the virus followed by incubation at 37 °C for 2 h for viral attachment to the cells. After virus attachment, the monolayers were washed and treated separately with 100 µL of a given antiviral. The titers of 229E were calculated after 5 days of incubation at 37 °C in a 5% CO_2_ incubator and compared with the control virus titer.

## 3. Results and Discussion

### 3.1. Molecular Docking Simulation on Mpro

As mentioned above, the binding affinity of over 17,000 compounds in the DrugBank database [29] was studied against the main protease (Mpro) of SARS-CoV-2 using crystallized protein (PDB: 6LU7 [27]). The selection of the best ligand-receptor docking complexes was based on the values of binding affinity reported as docking scores by the Glide module in the Schrödinger software. To assess the quality of the ligand-receptor interaction, the results of the simulation were compared with those obtained with co-crystallized peptide inhibitor N3 on 6LU7 [49]. The results of the docking study for each molecule of the DrugBank database along with its docking score (lower than −8 kcal/mol and higher than −2 kcal/mol) are shown in Tables S1 and S2 in the Supplementary Materials Section. Based on the results of the docking simulations, a pool of 274 molecules was created for use in the QSAR studies.

### 3.2. SARS-CoV-2 Main Protease QSAR Models and Validation

To identify potential drugs with anti-Mpro activity, four computational models based on different statistical techniques were developed including linear discriminant analysis (LDA), multilinear regression analysis (MLR), and artificial neural network (ANN) models for classification and regression analyses.

#### 3.2.1. Discriminant Models and Validation

The first discriminant QSAR model predicts the qualitative capacity of the drugs to interact with Mpro of SARS-CoV-2 and enable its inhibition. Compounds are classified as active or inactive if their docking scores are <−8 kcal/mol or >−2 kcal/mol, respectively. Both training and test data sets were used to construct each model. The first model, named “(DF_Class_6LU7_)”, focuses on identifying the mathematical pattern that discriminates between drugs with low and high docking scores against the protease. The second model was developed using the same data and machine learning methods. To be precise, artificial neural networks were used to generate a predictive model capable of classifying potential protease inhibitors based on the docking score values. Table 1 shows the statistics and descriptors for the two classification models. Detailed information regarding training and external test sets for classification models is given in Tables S1–S4.

Matrices for classification models showing an average correct classification rate of 97% for all models are shown in Table 2. Both DF_Class_6LU7_ and ANN_Class_6LU7_ models report higher sensitivity than specificity. However, its ability to identify inactive drugs is above 94%. The results obtained after external validation were even better than those presented by the selected models (Table 2); higher than 97% correct classification was found for test sets for both models indicating that the models are robust and reliable. The same classification matrices were obtained for training and test data sets of both models, which indicates that there is no difference between linear and non-linear statistical techniques to predict anti-SARS-CoV-2 activity by means of docking score value of 6LU7 protease.

**Figure 3:**
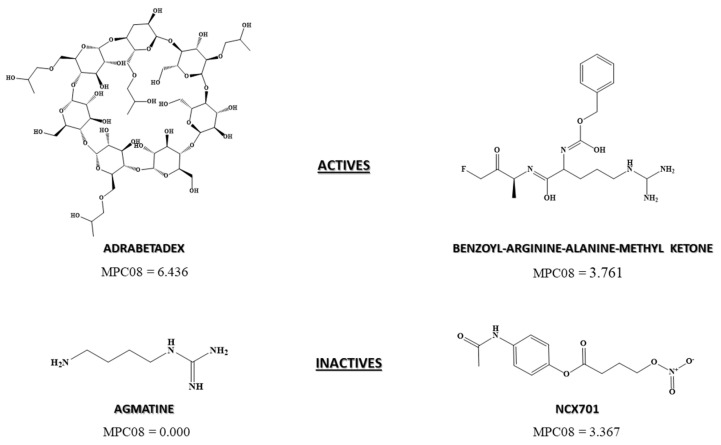
Differences in MPC08 value from active and inactive drugs as anti-SARS-CoV-2.

Both models were built using a path count descriptor (MPC08) implemented inalvaDesc version 2.0.6, Lecco (Italy), 2021 [40]. This index, obtained from a hydrogen-depleted molecular graph G, encodes the number of paths of order 8 that can be identified in a molecule. As this descriptor takes a positive value for DF_Class_6LU7_ equation, a higher number of paths of order 8 in a molecule results in a greater chance of exhibiting anti-SARS-CoV-2 activity. Figure 3 shows that compounds with MPC08 value greater than 3.7 are considered active by the DF_Class_6LU7_ model (for instance adrabetadex and benzoyl-arginine-alanine-methyl ketone) while those with lower values are labeled as inactive (agmatine and NCX701). Agmatine has a MPC08 value of zero, as no path of order 8 is present in its structure. It appears that there is an optimal number of paths of order 8 above which it is possible to establish a correlation with anti-SARS-CoV-2 activity. This is probably because NCX701 contains a path of order 8 in its structure, despite being labeled as inactive. This chemo-mathematical feature may also be correlated with biochemistry when considering a possible correlation with the steric space of the molecules inside the catalytic pocket of the protein. A higher volume of the ligands indicates a higher probability to establish interactions.

#### 3.2.2. Regression Models and Validation

Once the discriminant classification models were developed and validated, two other models were introduced in the QSAR strategy focused on the quantitative prediction of the most favorable docking score for potential 6LU7 protease inhibitors. Two QSAR regression models were built; a multilinear regression model MLR_reg_6LU7_ and an artificial neural network regression model ANN_reg_6LU7_. The same data sets used for the discriminant models (training and test set) were employed, but this time the objective was to predict the docking scores of the interactions with 6LU7 protease. Detailed information regarding training and external test sets for both MLR and ANN regression models is given in Tables S5–S8.

As seen in Table 3, both models yield similar correlation coefficients for the training and external test sets. Therefore, both linear and non-linear statistical techniques show good results when predicting the docking score. It should be noted that the ANN employs only four descriptors to train the network while the MLR model includes up to six descriptors in its equation. Nevertheless, considering the size of the dataset with 206 compounds, a 4- or 6-descriptor predictive model does not suffer from over-fitting. The descriptors used for the construction of regression models are listed in Table 4. It is evident that different types of indices are involved, such as 2D matrix-based descriptors, atom-centered fragments, chirality descriptors, edge adjacency indices, functional groups count, and pharmacophore descriptors. Therefore, our regression models take into account different aspects of the data set by using different types of descriptors implemented in the alvaDesc software version 2.0.6, Lecco (Italy). 2021 [40].

We then analyzed some of the most relevant descriptors for determining the chemico-mathematical pattern related to anti-protease activity by means of docking score prediction. Docking score predicted by the MLR model adopts negative values for drugs with theoretically higher anti-protease activity. Therefore, descriptors contributing negatively to the equation are *a priori* the ones with a direct impact in explaining the property: SpDiamEa(bo), Eig09EA(bo), nRNR2, and nLevel1. However, in the case of nLevel1, a direct correlation with docking score value between active and inactive drugs can be observed. As shown in Table 4, nLevel1 considers the number of neighboring atoms of the chiral center.

**Figure 4:**
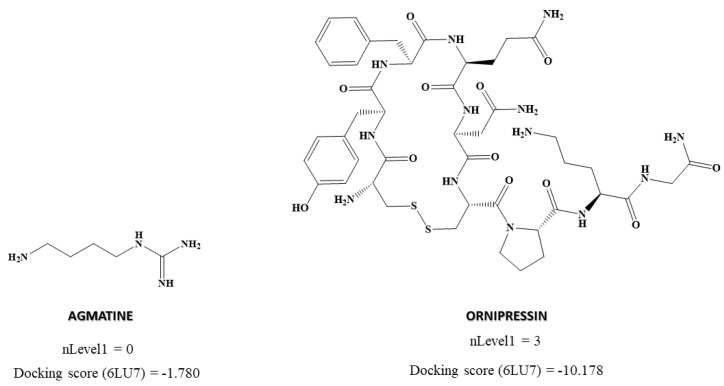
A descriptor directly correlated with the prediction of docking score of SARS-CoV-2 protease by MLR_reg_6LU7_.

Table S5 shows that almost all drugs with a low docking score value (potential protease inhibitors) exhibit a value of 3 on this descriptor. On the other hand, higher values of docking score (non-protease inhibitors) are related to ligands exhibiting 0 value at nLevel1 chirality descriptor do not have a chiral center. Figure 4 shows two drugs as an example of this pattern highlighted by the nLevel1 descriptor; Ornipressin exhibiting a low docking score value (potential protease inhibitors) and adopting a value of 3 on this descriptor, and Agmatine with a predicted higher docking score value (non-protease inhibitors) and adopting a 0 value for the nLevel1 descriptor.

Regression QSAR models (employing 2D descriptors) already described are focused on inferred molecular docking scores (determined by the 3D ligand conformation analysis), providing a rapid way to screen large databases of small molecules without conducting full molecular docking simulations. So why don’t we use 3D descriptors? The purpose of our QSAR analysis was only to predict docking scores capacity for unknown compounds (without considering the conformation adopted by the drug), as Daré and Freitas reported [50] the 2D approximation seems to be the optimal one, as it avoids the complex steps of conformational screening and 3D alignment while providing robust predictive models. However, if the scope of the QSAR model is explaining the effects of bioactive conformations in docking score then 3D descriptors could add valuable information to the prediction.

#### 3.2.3. ROC Curve

**Figure 5:**
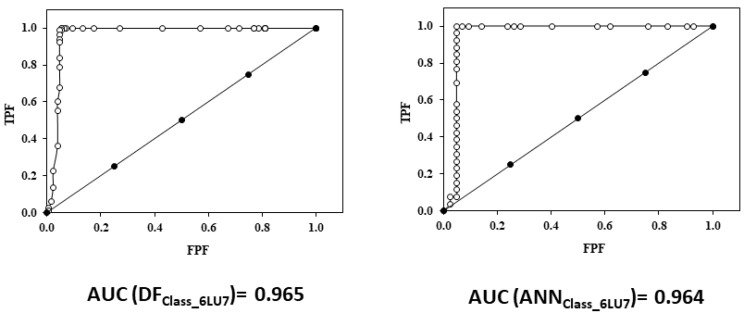
ROC curve for DF_Class_6LU7_ and ANN_Class_6LU7._ TPF: true positive fraction; FPF: false positive fraction.

To assess the reliability of the classification models, ROC curves were generated for each model. The ROC curve provides a graphical plot that illustrates the diagnostic ability of a binary classifier system as its discrimination threshold is varied. Figure 5 shows the ROC curves for LDA and ANN_Class_6LU7_ models. For this discriminant equation, the area under the curve (AUC) is greater than 0.96 for all models, which suggests a 96% chance that the models will correctly distinguish between an active and inactive/decoy compound.

#### 3.2.4. Pharmacological Distribution Diagram

**Figure 6:**
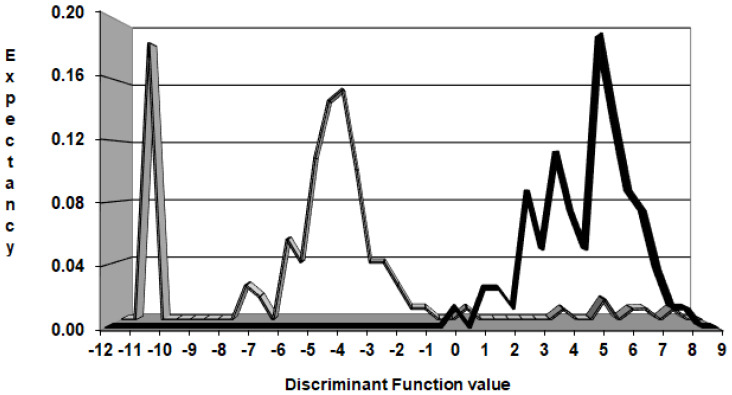
Pharmacological distribution diagram for anti-SARS-CoV-2 drugs.

The pharmacological distribution diagram (PDD) provides information on the applicability range of discriminant models. The PDD for the LDA model (Figure 6) indicates a very intuitive way for distribution and classification of the training set data (active and inactive). As shown in Figure 6, DF_Class_6LU7_ values between 1 and 8.25 show the range of the highest expectancy of harboring anti-SARS2 drugs. Drugs that do not present anti-SARS-CoV-2 activity, generally have DF values between −12 and 0 although we found some areas with slight overlapping between active and inactive throughout the PDD. Finally, drugs with DF values greater than 9 and less than −12 were considered outliers.

**Figure 7:**
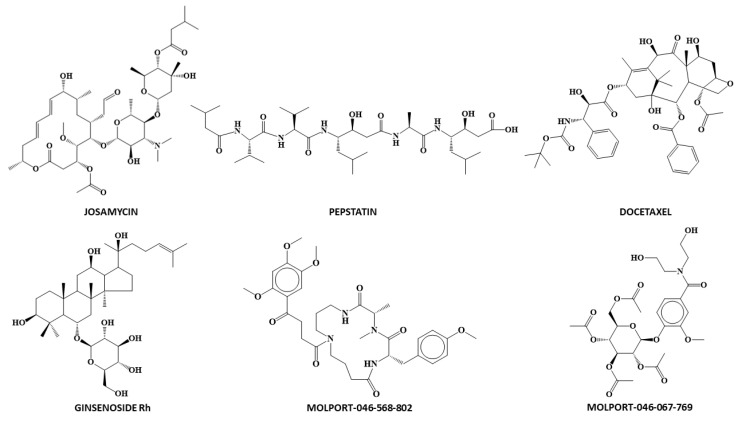
Chemical structure of the molecules (n = 6) selected during virtual screening.

Once the chemo-mathematical pattern of drugs is determined with docking score values against Mpro, it is possible to virtually screen for novel drugs that may have anti-SARS2 activity. The molecules with potential anti-SARS-2 activity (1) should be labeled as active by classification models (DF_Class_6LU7_ and ANN_Class_6LU7_); (2) should have a predicted docking score lower than −5 kcal/mol (MLR_reg_6LU7_ and/or ANN_reg_6LU7_), and finally (3) be commercially available. Molecular structures of the final pool of compounds selected by virtual screening are shown in Figure 7.

As shown in Table 5, six compounds were labeled as potential anti-SARS-CoV-2 agents, with a probability of activity or confidence level greater than 85% by the classification models and at least one regression model predicting a docking score value of −5 kcal/mol. However, Docetaxel and Ginsenoside showed a difference of one kcal/mol between MLR and ANN regression models. For the remaining four compounds both models predict the same value of the docking score. The values of the descriptors for the selected anti-SARS-CoV-2 compounds are shown in Table S9.

**Figure 8:**
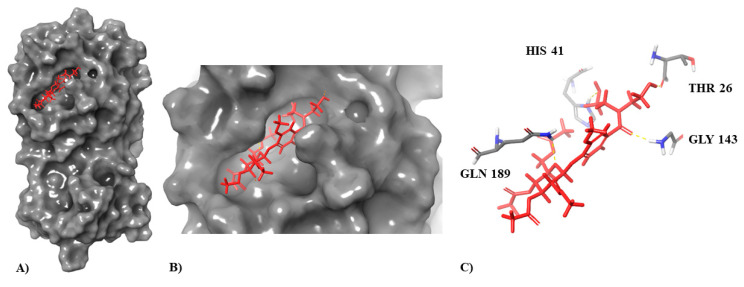
Docking pose (**A**,**B**) and amino acid interaction (**C**) of top-rank compound on 6LU7: Molport-046-067-769.

To further validate the developed predicted models, we performed a docking studio obtaining the theoretical docking score values of these compounds against SARS-CoV-2 protease (PDB: 6LU7). All six compounds had docking score values of <−4 kcal/mol (Table 6). Analyzing the co-crystallized ligand interactions with Mpro, it can be seen as the peptide inhibitor N3 interacted through hydrogen bonds with residues Thr26, Gln189, Glu166, Asn142, and Gly143 (Figure 2B) of 6LU7 with the depiction of the binding site (Figure 2A). Of all potential anti-SARS-CoV-2 compounds selected by Molecular Topology stands Molport-046-067-769 as the one with higher binding affinity for 6LU7 binding pocket (docking score: −7.514) (Figure 8), closest to co-crystallized ligand inhibitor N3 (docking score: −8.019). This compound establishes interactions with four of the five amino acids (AAs) with which the co-crystallized ligand interacts with Mpro (Inhibitor N3) named Thr26, Gln189, Glu166, and Gly143 (Table 6). All AA interactions with Mpro binding pocket from all reported potential anti-SARS-CoV-2 are reported in Table 6.

Finally, these six compounds were tested in vitro to further validate our models and to determine their ability as anti-SARS-CoV-2 compounds using human coronavirus 229E as a surrogate. It is worth noting that some of these compounds have already been reported as being antiviral and/or anti-COVID-19. For example, Ginsenoside has shown both antiviral [51,52,53] and anti-COVID-19 [54] activities. Josamycin is considered to have anti-COVID-19 activity [55,56] while Pepstatin A has been reported as antiviral [57,58,59] as well as a SARS-CoV-2 protease inhibitor [60,61,62]. As far as we know, no antiviral activity has been reported for Docetaxel, Molport-046-067-769, and Molport-046-568-802.

### 3.3. Antiviral Activity of Chemicals against Human Coronavirus 229E

The human coronavirus 229E was used as a surrogate of SARS-CoV-2. The virus was grown and titrated in MRC-5 cells (human lung fibroblast cells). Before starting the experiment, all six compounds were dissolved individually in 100% DMSO at a concentration of 500 µg/mL. This solution was then diluted 1:2 (250 µg/mL), 1:5 (100 µg/mL), and 1:10 (50 µg/mL) followed by testing the cytotoxicity of all four concentrations in MRC-5 cells. We observed that solutions containing 500 µg/mL, 250 µg/mL, and 100 µg/mL of all six compounds were toxic to MRC-5 cells. Hence, further in vitro testing of antiviral activity was done using 50 µg/mL of the compounds. Since it was a proof-of-the-concept study, we tested only a single concentration of the six compounds against human coronavirus 229E. The results shown in Table 7 are an average of duplicate experiments.

As shown in Table 7, all compounds inhibited >99% of the human coronavirus 229E when administered as pre-treatment (treatment of cells with the compound before applying virus) and co-treatment (treatment of cells with indicated compound and the virus simultaneously). In the post-treatment modality (treatment of cells with the compound after application of the virus), however, 90–99% of the virus was inactivated. Docetaxel is a well-known antimitotic agent, derived from paclitaxel, with a significant level of toxicity. Its common adverse reactions include a reduction in immune system activity and certain blood disorders. Josamycin is a macrolide with an average safety profile in terms of toxicity. However, there are reports of complications related to its use in the presence of hepatic or renal impairment. In addition, there is a potential for cardiotoxicity related to prolongation of the QT interval, although it is seldom fatal. Pepstatine A seems to be a valid candidate, with acceptable levels of toxicity. The same is true for Ginsenoside rh1, which is essentially safe and is of natural origins. As far as we know, the anti-SARS-CoV-2 action of Molport-046-067-769 and Molport-046-568-802 is reported here for the first time. These two compounds do not appear to have any significant side effects or toxicity.

## 4. Conclusions

This study reports the identification of a set of potential inhibitors of the main protease of SARS-CoV-2 in silico and in vitro. We achieved this by using three-dimensional information, e.g., molecular docking, ligand-receptor interactions into chemometrics terms, and by using pattern recognition and machine learning techniques based on molecular topology. The idea was to show how the topological paradigm, which is capable of translating physicochemical information into a set of numbers by means of graph theory, can be a useful and time-saving tool in drug design and discovery. Despite the known ability of molecular docking to determine specific ligand-receptor interaction, screening a large number of molecules against a specific receptor is usually a long process. The possibility to translate this process, into a set of topological models based on discriminant and regression analysis, allows the screening of larger databases (billions of molecules) in a very short time. Thanks to the QSAR strategy, the in silico results were robust, with an average rate of correct classification for the discriminant analysis above 97% for both active and inactive compounds. The best candidates selected during the computational strategy were then tested in vitro on human coronavirus 229E. All compounds showed >99% virus inhibition when administered as pre-treatment and co-treatment and values of 90–99% of inhibition when administered as post-treatment. Further studies are needed for a deeper analysis of the selected molecules to determine which of them may be the most suitable for repurposing against SARS-CoV-2. Several factors such as price, toxicity, and potential risks (e.g., generation of antiviral resistance) need to be studied. Both Molport-046-067-769 and Molport-046-568-802 are described for the first time here as potential inhibitors of SARS-CoV-2 main protease. In addition, according to the literature, their toxicity profile is favorable. Thus, we believe that both of them could be good candidates for further testing as potential treatments against SARS-CoV-2.

## Figures and Tables

**Table 1:** Development of classification models to predict the anti-SARS-CoV-2 activity (protease inhibition activity).

Statistical Method	Model	Model Parameters
LDA	$DF_{Class\_6LU7}=\left(2.917\times MPC08\right)-10.550$	N = 206 λ = 0.305 F = 417.37 *p* < 0.00001
ANN	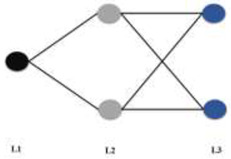 **ANN_Class_6LU7_** MLP 1 *-2-2	Training algorithm: BFGS 8 Error function: Entropy Hidden activation function: Tanh Output activation function: Softmax

N: number of molecules; λ: Wilks’ lambda; F: Fischer-Snedecor parameter; p: p-value or probability value. * Input network: MPC08.

**Table 2:** Classification matrices for classification models and external validation.

		Model			External Validation		
		% of Correct Classification	Active	Inactive	% of Correct Classification	Active	Inactive
	Active	100.0	80	0	100.0	26	0
**DF_Class_6LU7_**	Inactive	94.4	7	119	95.2	2	40
	Average	97.2			97.6		
	Active	100.0	80	0	100.0	26	0
**ANN_Class_6LU7_**	Inactive	94.4	7	119	95.2	2	40
	Average	97.2			97.6		

**Table 3:** Regression models developed to predict docking score against protease 6LU7 of SARS-CoV-2.

Statistical Method	Model	Model Parameters
MLR	$\begin{array}{cc} Docking\;score\; & \left(6LU7\right)\\ & =1.041\\ & -\left(0.614\times {\mathrm{SpDiam}}_{\mathrm{EA}\left(\mathrm{bo}\right)}\right)\\ & -\left(1.765\times \mathrm{Eig}09_{\mathrm{EA}\left(\mathrm{bo}\right)}\right)\\ & -\left(6.403\times \mathrm{nRNR}2\right)\\ & +\left(7.069\times N-068\right)\\ & +\left(0.115\times \mathrm{CATS}2D_{05_{\mathrm{LL}}}\right)\\ & -\left(0.463\times \mathrm{nLevel}1\right) \end{array}$	N = 206 r^2^ = 0.884 F = 255.927 *p* < 0.00001 SEE = 1.415 q^2^ = 0.741
ANN	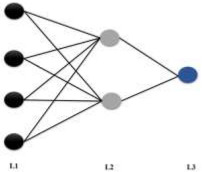 ANN_reg_6LU7_ MLP 4 *-2-1	N = 206 r^2^ = 0.887 q^2^ = 0.764 Training algorithm: BFGS 73 Error function: SOS Hidden activation function: Tanh Output activation function: Logistic

r^2^: correlation coefficient; SEE: Standard error of estimate; q^2^, cross-validation correlation coefficient. * Input network: SM4_B(m), Eig09_EA(bo), CATS2D_05_LL, s2_relPathLength.

**Table 4:** Topo-chemical descriptors used in the construction of SARS-CoV-2 models.

Descriptor Type	Descriptor Name	Descriptor Definition
2D matrix-based descriptors	SM4_B(m)	Spectral moment of order 4 from Burden matrix weighted by mass
Atom-centered fragments	N-068	Al3-N
Chirality descriptors	nLevel1	Number of neighboring atoms of the chiral center (level 1)
Chirality descriptors	s2_relPathLength	Maximum path length of the substituent 2 normed by the heavy atoms
Edge adjacency indices	Eig09_EA(bo)	Eigenvalue nº 9 from edge adjacency matrix weighted by bond order
Edge adjacency indices	SpDiam_EA(bo)	Spectral diameter from edge adjacency matrix weighted by bond order
Functional group counts	nRNR2	Number of tertiary amines (aliphatic)
Pharmacophore descriptors	CATS2D_05_LL	CATS2D Lipophilic-Lipophilic at lag 05

**Table 5:** Six drugs selected by the Molecular Topology strategy as potential anti-SARS-CoV-2.

Drug	DF_Class_6LU7_	P.A.	ANN_Class_6LU7_	Conf. Levels	Docking ScoreMLR_reg_6LU7_	Docking ScoreANN_reg_6LU7_
Docetaxel	7.630	1.000	1	0.873	−7.853	−6.813
Ginsenoside	8.379	1.000	1	0.873	−5.319	−4.300
Josamycin	5.473	0.996	1	0.873	−6.158	−6.718
Molport-046-067-769	4.598	0.990	1	0.872	−8.628	−8.842
Molport-046-568-802	3.557	0.972	1	0.871	−8.213	−8.584
Pepstatin A	1.758	0.853	1	0.854	−6.163	−5.975

Conf. confidence; P.A.: probability of being classified as active by the LDA model. Molport-046-067-769:[(3R,6S)-3,4,5-tris(acetyloxy)-6-{4-[bis(2-hydroxyethyl)carbamoyl]-2-methoxyphenoxy}oxan-2-yl]methylacetate; Molport-046-568-802:(2S,5S)-2-[(4-methoxyphenyl)methyl]-4,5-dimethyl-11-[4-oxo-4-(2,4,5- trimethoxyphenyl)butanoyl]-1,4,7,11-tetraazacyclopentadecane-3,6,15-trione.

**Table 6:** Potential anti-SARS-CoV-2 compounds selected by Molecular Topology and docking score for Mpro (PDB:6LU7).

Compound	Docking Score	Interaction with Indicated Amino Acids
Inhibitor N3 (co-crystallized ligand)	−8.019	Glu166 (3× H, salt bridge) Gly143 (H) Thr26 (H) Asn142(2× H) Gln189 (H)
Molport-046-067-769	−7.514	Glu166 (2× H, salt bridge) Gly143 (H) Thr26 (H) Gln189 (H) His41 (H)
Pepstatin A	−7.155,	Glu166 (2× H, salt bridge) Gln189 (H) His164 (H) Ala191(H)
Docetaxel	−6.916	Glu166 (4× H, salt bridge) Gln189 (H)
Molport-046-568-802	−6.361	Glu166 (H) Asn142 (H) Thr26 (H) Gln189 (H)
Ginsenoside	−5.319	Gln189 (H) Glu166 (2× H) Gly170(H)
Josamycin	−3.995	Glu166 (2× H, salt bridge)

**Table 7:** The effect of 50ug/mL of the six compounds on human coronavirus 229E.

Compound	Virus Titers Shown as Log_10_ TCID_50_/100 µL (Per Cent Virus Inactivation)			
	Stock Virus	Pre-Treatment	Co-Treatment	Post-Treatment
Josamycin	5.7	2.83 (99.87)	3.1 (99.75)	4.0 (98.00)
Pepstatin	5.7	3.5 (99.37)	3.6 (99.20)	4.5 (93.69)
Docetaxel	5.5	3.0 (99.68)	3.5 (99.00)	4.5 (90.00)
Molport-046-067-769	5.5	2.83 (99.78)	2.60 (99.87)	3.83 (97.86)
Molport-046-568-802	5.5	2.66 (99.85)	3.16 (99.54)	4.1 (96.01)
Ginsenocide Rh1	5.5	2.83 (99.78)	2.05 (99.96)	3.5 (99.00)

## Data Availability

The main data presented in this study is contained within the Supplementary Materials to this article.

## Supplementary Materials

The following supporting information can be downloaded at: https://www.mdpi.com/article/10.3390/biomedicines10061342/s1, Table S1: DFClass_6LU7 training set: compounds identification, 6LU7 Mpro docking score, descriptors value, classification and probability of being classified as active; Table S2: DFClass_6LU7 external set: compounds identification, 6LU7 Mpro docking score, descriptors value, classification and probability of being classified as active; Table S3: ANNClass_6LU7 training set: compounds identification, 6LU7 Mpro docking score, descriptors value, classification and confidence level associated; Table S4: ANNClass_6LU7 external set: compounds identification, 6LU7 Mpro docking score, descriptors value, classification and confidence level associated; Table S5: MLRA_reg6LU7 training set: compounds identification, 6LU7 Mpro docking score, descriptors value and 6LU7 Mpro docking score predicted; Table S6: MLRA_reg6LU7 external set: compounds identification, 6LU7 Mpro docking score, descriptors value and 6LU7 Mpro docking score predicted; Table S7: ANNreg_6LU7 regression training set: compounds identification, 6LU7 Mpro docking score, descriptors value and 6LU7 Mpro docking score predicted; Table S8: ANNreg_6LU7 regression external set: compounds identification, 6LU7 Mpro docking score, descriptors value and 6LU7 Mpro docking score predicted; Table S9: Descriptors’ value for the selected anti-SARS-CoV-2 compounds.
