# Supplementary material for: COVID-19 and the Importance of Being Prepared: A Multidisciplinary Strategy for the Discovery of Antivirals to Combat Pandemics
Source: Biomedicines. 2022 Jun 7;10(6):1342. doi: 10.3390/biomedicines10061342 (PMC9220014; doi:10.3390/biomedicines10061342)
Supplement: Supplementary file 1 [file biomedicines-10-01342-s001.zip › biomedicines-1723488-supplementary.pdf]

COVID-19 and the importance of being prepared: A multidisciplinary strategy for the discovery of antivirals to combat pandemics

Maria Galvez-Llompart, Riccardo Zanni, Jorge Galvez, Subhash C. Basak, Sagar M. Goyal

Supplementary Material

Table S1. DF_{Class_6LU7} training set: compounds identification, 6LU7 Mpro docking score, descriptors value, classification and probability of being classified as active.

Compound	Drugbank id.	Drugbank category	Docking score (6LU7)	MPC08	DF_{Class_6UL7}	Class.	P.A.
**Active group**							
Gonadorelin	DB00644	Approved; Invest.; Vet	-11.695	5.684	6.029	A	0.998
Ornipressin	DB13464	Exper	-11.367	5.609	5.813	A	0.997
Felypressin	DB00093	Exper	-10.940	4.956	3.906	A	0.980
Carbetocin	DB01282	Approved; Invest.	-10.381	5.380	5.143	A	0.994
(E)-(4S,6S)-6-((S)-2-{(S)-2-[(furan-2-carbonyl)-amino]-3-methyl-butyrylamino}-3-methyl-butyrylamino)-8-methyl-5-oxo-4-((r)-2-oxo-pyrrolidin-3-ylmethyl)-non-2-enoic acid ethyl ester	DB04613	Exper	-10.222	5.897	6.652	A	0.999
Iotrolan	DB09487	Approved	-10.176	4.605	2.883	A	0.947
Lypressin	DB14642	Approved	-10.107	5.513	5.533	A	0.996
Triptorelin	DB06825	Approved; Vet	-10.045	5.945	6.793	A	0.999
Lanreotide	DB06791	Approved	-9.973	4.956	3.906	A	0.980
Birinapant	DB11782	Invest.	-9.893	6.084	7.198	A	0.999
Indium In-111 pentetreotide	DB11835	Approved; Invest.	-9.877	6.116	7.290	A	0.999
Ipamorelin	DB12370	Invest.	-9.873	4.754	3.316	A	0.965
UK-432097	DB12691	Invest.	-9.697	5.756	6.240	A	0.998
Pentagastrin	DB00183	Approved	-9.648	4.984	3.987	A	0.982
Terlipressin	DB02638	Approved; Invest.	-9.511	6.192	7.513	A	0.999
Succinamide-Coa	DB03905	Exper	-9.472	5.493	5.473	A	0.996
Iopamidol	DB08947	Approved	-9.397	5.464	5.388	A	0.995
BV1	DB04210	Exper	-9.387	5.549	5.637	A	0.996
Labradimil	DB06549	Invest.	-9.377	5.371	5.116	A	0.994
Etelcalcetide	DB12865	Approved; Invest.	-9.353	4.942	3.865	A	0.979
LY231514 Tetra Glu	DB02223	Exper	-9.318	4.812	3.487	A	0.970
talactoferrin alpha	DB05426	Invest.	-9.217	5.333	5.006	A	0.993

8-Demethyl-8-Dimethylamino-Flavin-Adenine-Dinucleotide	DB03482	Exper	-9.206	4.533	2.672	A	0.935
N-(Sulfanylacetyl)Tyrosylprolylmethioninamide	DB01883	Exper	-9.162	3.970	1.031	A	0.737
Colistin	DB00803	Approved	-9.125	5.357	5.075	A	0.994
4'-nitrophenyl-3i-thiolaminaritrioside	DB03990	Exper	-9.014	5.858	6.538	A	0.999
Hydroxyethyl cellulose	DB11602	Approved	-9.014	5.684	6.029	A	0.998
S-(2-Oxo)Pentadecylcoa	DB02271	Exper	-9.000	5.069	4.236	A	0.986
4-Hydroxyphenacyl Coenzyme A	DB03613	Exper	-8.999	5.416	5.249	A	0.995
Coa-S-Acetyl Tryptamine	DB02931	Exper	-8.927	5.541	5.614	A	0.996
Vapreotide	DB04894	Exper; Invest.	-8.904	5.762	6.258	A	0.998
Adrabetadex	DB15146	Invest.	-8.894	6.436	8.224	A	1.000
Thymopentin	DB11996	Invest.	-8.889	4.554	2.734	A	0.939
Saquinavir	DB01232	Approved; Invest.	-8.844	5.403	5.210	A	0.995
3-Thiaoctanoyl-Coenzyme A	DB03415	Exper	-8.745	4.007	1.139	A	0.758
4-methyl-pentanoic acid {1-[4-guanidino-1-(thiazole-2-carbonyl)-butylcarbamoyl]-2-methyl-propyl}-amide	DB07299	Exper	-8.745	5.580	5.726	A	0.997
Sinapoyl Coenzyme A	DB03179	Exper	-8.736	4.984	3.987	A	0.982
P1-(5'-Adenosyl)P5-(5'-Thymidyl)Pentaphosphate	DB03280	Exper	-8.726	4.500	2.576	A	0.929
Dotatate	DB14554	Approved; Exper	-8.708	5.509	5.521	A	0.996
3-[3-(2,3-Dihydroxy-Propylamino)-Phenyl]-4-(5-Fluoro-1-Methyl-1h-Indol-3-Yl)-Pyrrole-2,5-Dione	DB01772	Exper	-8.697	5.063	4.218	A	0.985
Iobitridol	DB12407	Approved; Invest.	-8.697	5.112	4.362	A	0.987
Angiotensin II	DB11842	Approved; Invest.	-8.674	5.485	5.449	A	0.996
Flavin adenine dinucleotide	DB03147	Approved	-8.539	5.476	5.425	A	0.996
Iodixanol	DB01249	Approved	-8.538	5.017	4.085	A	0.983
Sufugolix	DB06494	Invest.	-8.501	4.234	1.801	A	0.858
Elamipretide	DB11981	Invest.	-8.476	5.768	6.276	A	0.998
Flavin-N7 Protonated-Adenine Dinucleotide	DB02332	Exper	-8.446	5.976	6.883	A	0.999
Alatrofloxacin	DB09335	Approved; Withdrawn	-8.428	5.704	6.088	A	0.998
Methylmalonyl-Coenzyme A	DB04045	Exper	-8.428	5.004	4.047	A	0.983
Coa-S-Trimethylene-Acetyl-Tryptamine	DB01777	Exper	-8.422	5.513	5.533	A	0.996
Trifluoroacetonyl Coenzyme A	DB01969	Exper	-8.388	5.624	5.855	A	0.997
Je-2147, Ag1776, Kni-764	DB02668	Exper	-8.329	5.198	4.614	A	0.990
Giripladib	DB15426	Invest.	-8.314	4.779	3.391	A	0.967

Efrotomycin	DB11401	Vet	-8.288	4.615	2.912	A	0.948
3-[1-(3-aminopropyl)-1h-indol-3-yl]-4-(1h-indol-3-yl)-1h-pyrrole-2,5-dione	DB07457	Exper	-8.259	5.347	5.048	A	0.994
Fosifloxuridine nafalbenamide	DB14859	Invest.	-8.250	5.268	4.816	A	0.992
HM-30181	DB14070	Exper	-8.238	4.466	2.477	A	0.923
2-Hydroxy-5-({1-[(4-Methylphenoxy)Methyl]-3-Oxoprop-1-Enyl}Amino)-L-Tyrosine	DB02537	Exper	-8.232	4.277	1.925	A	0.873
Caspofungin	DB00520	Approved	-8.208	4.927	3.823	A	0.979
Benzoyl-Arginine-Alanine-Methyl Ketone	DB03536	Exper	-8.200	3.761	0.421	A	0.604
Inhibitor Bea388	DB04255	Exper	-8.191	5.493	5.473	A	0.996
3-[(5s)-1-Acetyl-3-(2-Chlorophenyl)-4,5-Dihydro-1h-Pyrazol-5-Yl]Phenol	DB03996	Exper	-8.186	5.380	5.143	A	0.994
Cefbuperazone	DB13638	Exper	-8.171	4.500	2.576	A	0.929
Telinavir	DB12178	Invest.	-8.164	5.961	6.838	A	0.999
Nicotinamide-Adenine-Dinucleotide-5-Hydroxy-4-Oxonorvaline	DB02349	Exper	-8.154	5.609	5.813	A	0.997
Cangrelor	DB06441	Approved	-8.151	5.557	5.659	A	0.997
Polymyxin B	DB00781	Approved; Vet	-8.149	5.489	5.461	A	0.996
N-(4-carbamimidoylbenzyl)-1-(4-methylpentanoyl)-L-prolinamide	DB06936	Exper	-8.129	5.338	5.020	A	0.993
NUC-1031	DB15057	Invest.	-8.118	4.949	3.886	A	0.980
Larazotide	DB05645	Invest.	-8.117	5.533	5.591	A	0.996
Inarigivir soproxil	DB15063	Invest.	-8.105	5.677	6.009	A	0.998
Cephalosporin Analog	DB02136	Exper	-8.077	5.308	4.934	A	0.993
FR236913	DB02830	Exper	-8.056	5.176	4.549	A	0.990
TOP-1288	DB14839	Invest.	-8.054	4.883	3.693	A	0.976
Ioforminol	DB12439	Invest.	-8.050	4.762	3.341	A	0.966
Dirlotapide	DB11399	Invest.; Vet	-8.034	4.898	3.737	A	0.977
BMS-986094	DB11966	Invest.	-8.021	6.033	7.049	A	0.999
Iopentol	DB13861	Exper	-8.016	4.543	2.703	A	0.937
Carfilzomib	DB08889	Approved; Invest.	-8.009	5.056	4.199	A	0.985
4-Oxo-Nicotinamide-Adenine Dinucleotide Phosphate	DB01753	Exper	-8.002	5.919	6.715	A	0.999
**Inactive Group**							
Agmatine	DB08838	Exper; Invest.	-2.000	0.000	-10.550	I	0.000
1-acetyl-2-lyso-sn-glycero-3-phosphoethanolamine	DB04731	Exper	-1.961	2.398	-3.555	I	0.028
Glycerin	DB09462	Approved; Invest.	-1.961	0.000	-10.550	I	0.000

Magnesium glycinate	DB11189	Approved	-1.960	0.000	-10.550	I	0.000
Zinc glycinate	DB14493	Exper	-1.960	0.000	-10.550	I	0.000
Aluminum zirconium octachlorohydrex gly	DB11200	Approved	-1.956	0.000	-10.550	I	0.000
Dihydroxyacetone	DB01775	Exper	-1.956	0.000	-10.550	I	0.000
Ferrous glycine sulfate	DB14501	Approved	-1.956	0.000	-10.550	I	0.000
(S)-2-Amino-4-[(2s,3r)-2,3,5-Trihydroxy-4-Oxo-Pentyl]Mercapto-Butyric Acid	DB04182	Exper	-1.908	2.485	-3.302	I	0.036
1,4-Butanediol	DB01955	Exper	-1.859	0.000	-10.550	I	0.000
Amyl Nitrite	DB01612	Approved	-1.822	0.000	-10.550	I	0.000
3-Oxo-Pentadecanoic Acid	DB04039	Exper	-1.815	2.398	-3.555	I	0.028
4-Hydroxybutan-1-Aminium	DB02541	Exper	-1.809	0.000	-10.550	I	0.000
NB-001	DB12716	Invest.	-1.783	3.807	0.554	A	0.635
L-cystein-s-1-(iminomethyl)-l-ornithine	DB04671	Exper	-1.733	2.485	-3.302	I	0.036
L-Alpha-Glycerophosphorylserine	DB02497	Exper	-1.729	2.079	-4.484	I	0.011
2-Hydroxyethyl Disulfide	DB02486	Exper	-1.698	0.000	-10.550	I	0.000
Ricinoleic Acid	DB02955	Exper	-1.612	2.639	-2.852	I	0.055
cis-Vaccenic acid	DB04801	Exper	-1.610	2.565	-3.068	I	0.044
Tylosin	DB11475	Vet	-1.587	5.568	5.693	A	0.997
Dexpanthenol	DB09357	Approved	-1.578	1.609	-5.855	I	0.003
Nz-(1-Carboxyethyl)-Lysine	DB02370	Exper	-1.566	2.197	-4.141	I	0.016
Troleandomycin	DB13179	Approved	-1.566	5.513	5.533	A	0.996
N-(Phosphonoacetyl)-L-Ornithine	DB02011	Exper	-1.542	2.303	-3.833	I	0.021
Sevelamer	DB00658	Approved	-1.522	0.000	-10.550	I	0.000
3,7,11,15-tetramethyl-hexadecan-1-ol	DB01637	Exper; Invest.	-1.515	2.708	-2.651	I	0.066
Nz-(Dicarboxymethyl)Lysine	DB01815	Exper	-1.508	2.398	-3.555	I	0.028
L-Alpha-Glycerophosphorylethanolamine	DB03484	Exper	-1.502	1.386	-6.506	I	0.001
1,3-Propandiol	DB02774	Exper	-1.482	0.000	-10.550	I	0.000
N-Alpha-L-Acetyl-Arginine	DB01985	Exper	-1.475	2.197	-4.141	I	0.016
Methylethylamine	DB02396	Exper	-1.437	0.000	-10.550	I	0.000
(9Z,11E,13S)-13-hydroxyoctadeca-9,11-dienoic acid	DB06926	Exper	-1.410	2.639	-2.852	I	0.055
(3R)-3-hydroxydodecanoic acid	DB07930	Exper	-1.409	2.944	-1.961	I	0.123
N-Butyl-N'-Hydroxyguanidine	DB02727	Exper	-1.371	0.000	-10.550	I	0.000
2-Decenoyl N-Acetyl Cysteamine	DB03813	Exper	-1.357	2.398	-3.555	I	0.028
4-amino-n-[(2-sulfanylethyl)carbamoyl]benzenesulfonamide	DB08484	Exper	-1.335	2.833	-2.286	I	0.092

Cimetidine	DB00501	Approved; Invest.	-1.306	2.773	-2.462	I	0.079
undecylamine-n,n-dimethyl-n-oxide	DB07646	Exper	-1.302	2.079	-4.484	I	0.011
MF268	DB04021	Exper	-1.288	2.944	-1.961	I	0.123
Triethoxycaprylylsilane	DB11267	Exper	-1.283	2.398	-3.555	I	0.028
Ethyl Isocyanide	DB03399	Exper	-1.203	0.000	-10.550	I	0.000
Alpha-linolenic acid	DB00132	Approved; Invest.; Nutraceutical	-1.198	2.565	-3.068	I	0.044
Sodium lauryl sulfate	DB00815	Approved	-1.178	2.303	-3.833	I	0.021
Dodecyl sulfate	DB03967	Exper	-1.174	2.303	-3.833	I	0.021
Magnesium stearate	DB14077	Invest.	-1.174	3.219	-1.161	I	0.239
Guanidine-3-Propanol	DB03637	Exper	-1.153	0.000	-10.550	I	0.000
Dolastatin 10	DB12730	Invest.	-1.137	5.030	4.124	A	0.984
(10E,12Z)-octadecadienoic acid	DB04746	Exper	-1.114	2.565	-3.068	I	0.044
S-(D-Carboxybutyl)-L-Homocysteine	DB02337	Exper	-1.108	2.079	-4.484	I	0.011
N2-(Carboxyethyl)-L-Arginine	DB04189	Exper	-1.104	2.398	-3.555	I	0.028
diethyl propane-1,3-diylbiscarbamate	DB08501	Exper	-1.072	2.197	-4.141	I	0.016
GW-274150	DB12237	Invest.	-1.060	1.946	-4.874	I	0.008
Etoglucid	DB13339	Exper	-1.057	2.708	-2.651	I	0.066
Palmitoleic Acid	DB04257	Exper	-1.019	2.398	-3.555	I	0.028
Odalasvir	DB13041	Invest.	-1.013	6.526	8.488	A	1.000
3-Hydroxy-Myristic Acid	DB02767	Exper	-1.002	2.303	-3.833	I	0.021
Nitrosoethane	DB02646	Exper	-0.974	0.000	-10.550	I	0.000
Elaidoylamide	DB03784	Exper	-0.953	2.565	-3.068	I	0.044
Bromo-Dodecanol	DB02619	Exper	-0.947	1.946	-4.874	I	0.008
Ethylhexylglycerin	DB14557	Exper	-0.914	1.792	-5.323	I	0.005
Rifampicin	DB01045	Approved	-0.888	5.958	6.831	A	0.999
1-Guanidinium-7-Aminoheptane	DB03639	Exper	-0.881	1.609	-5.855	I	0.003
Ethanolamine oleate	DB06689	Approved	-0.866	2.565	-3.068	I	0.044
Ethanol	DB00898	Approved	-0.776	0.000	-10.550	I	0.000
C31G	DB05398	Invest.	-0.753	2.996	-1.811	I	0.140
n-dodecyl-n,n-dimethylglycinate	DB07631	Exper	-0.753	2.485	-3.302	I	0.036
5-N-Allyl-Arginine	DB03892	Exper	-0.752	2.197	-4.141	I	0.016
Hexaminolevulinate	DB06261	Approved	-0.718	2.079	-4.484	I	0.011
Cetrimonium	DB01718	Approved	-0.607	2.565	-3.068	I	0.044
Dibutylsuccinate	DB13332	Exper	-0.599	2.197	-4.141	I	0.016
2-[3-(2-Hydroxy-1,1-Dihydroxymethyl-Ethylamino)-Propylamino]-2-Hydroxymethyl-Propane-1,3-Diol	DB02676	Exper	-0.567	2.773	-2.462	I	0.079

Tiadenol	DB13348	Exper	-0.524	2.398	-3.555	I	0.028
N5-(1-Imino-3-Butenyl)-L-Ornithine	DB03710	Exper	-0.514	2.079	-4.484	I	0.011
3,6,9,12,15-Pentaoxaheptadecane	DB02343	Exper	-0.513	2.303	-3.833	I	0.021
N-Omega-Propyl-L-Arginine	DB02644	Exper	-0.511	2.197	-4.141	I	0.016
Dioctyldimonium	DB13970	Approved; Exper	-0.501	2.639	-2.852	I	0.055
Decamethonium	DB01245	Approved	-0.459	2.398	-3.555	I	0.028
11-[(mercaptocarbonyl)oxy]undecanoic acid	DB08712	Exper	-0.453	2.303	-3.833	I	0.021
N-Omega-Hydroxy-L-Arginine	DB03144	Exper	-0.414	1.946	-4.874	I	0.008
Dodecane-Trimethylamine	DB02779	Exper	-0.410	2.197	-4.141	I	0.016
Hydroxybutyloxide	DB14079	Exper	-0.395	1.609	-5.855	I	0.003
Tris(Hydroxyethyl)Aminomethane	DB04237	Exper	-0.364	0.000	-10.550	I	0.000
Lauroyl chloride	DB14670	Exper	-0.358	1.946	-4.874	I	0.008
12-Hydroxydodecanoic Acid	DB03704	Exper	-0.348	2.079	-4.484	I	0.011
Diethylhomospermine	DB13011	Invest.	-0.337	1.609	-5.855	I	0.003
Oleic Acid	DB04224	Approved; Invest.; Vet	-0.319	2.565	-3.068	I	0.044
Silanol	DB11343	Approved	-0.311	0.000	-10.550	I	0.000
N3, N4-Dimethylarginine	DB02302	Exper	-0.295	2.197	-4.141	I	0.016
Pentaglyme	DB02580	Exper	-0.221	2.398	-3.555	I	0.028
Ocrylate	DB15086	Invest.	-0.198	2.079	-4.484	I	0.011
Isopropyl myristate	DB13966	Approved; Exper	-0.188	2.485	-3.302	I	0.036
Monoctanoin	DB06801	Exper	-0.151	2.079	-4.484	I	0.011
3,6,9,12,15,18-hexaoxaicosane	DB06867	Exper	-0.112	2.565	-3.068	I	0.044
Vinyl ether	DB13690	Exper	-0.108	0.000	-10.550	I	0.000
NCX 701	DB05409	Invest.	-0.094	3.367	-0.728	I	0.326
Trolnitrate	DB13719	Exper	-0.094	2.773	-2.462	I	0.079
Methyl Nonanoate (Ester)	DB01631	Exper	-0.084	1.609	-5.855	I	0.003
Palmitic Acid	DB03796	Approved	-0.081	2.398	-3.555	I	0.028
sebacic acid	DB07645	Exper	-0.070	1.946	-4.874	I	0.008
Azelaic acid	DB00548	Approved	-0.064	1.792	-5.323	I	0.005
Bombykol	DB02982	Exper	-0.004	2.303	-3.833	I	0.021
Nitrous acid	DB09112	Approved; Invest.	0.022	0.000	-10.550	I	0.000
L-Homoarginine	DB03974	Exper	0.040	1.946	-4.874	I	0.008
O-Decyl Hydrogen Thiocarbonate	DB08684	Exper	0.106	1.946	-4.874	I	0.008
Lauric acid	DB03017	Approved; Exper	0.148	1.946	-4.874	I	0.008
Spermine	DB00127	Exper; Nutraceutical	0.161	1.946	-4.874	I	0.008

Quaternium-24	DB13969	Exper	0.164	2.773	-2.462	I	0.079
Rifapentine	DB01201	Approved; Invest.	0.328	6.019	7.006	A	0.999
Tetraglyme	DB14000	Exper	0.353	2.079	-4.484	I	0.011
3,6,9,12,15-pentaoxaheptadecan-1-ol	DB07344	Exper	0.471	2.398	-3.555	I	0.028
5-(2-hydroxyethyl)nonane-1,9-diol	DB07171	Exper	0.541	1.792	-5.323	I	0.005
Triglyme	DB02078	Exper	0.620	1.609	-5.855	I	0.003
decane-1-thiol	DB07611	Exper	0.704	1.386	-6.506	I	0.001
tetrabutylammonium ion	DB01851	Exper	0.711	1.946	-4.874	I	0.008
undecylenic acid	DB11117	Approved; Invest.	0.719	1.792	-5.323	I	0.005
1-dodecanol	DB06894	Exper	0.953	1.792	-5.323	I	0.005
bis(hexamethylene)triamine	DB04684	Exper	0.953	2.079	-4.484	I	0.011
N-ethyl-N-[3-(propylamino)propyl]propane-1,3-diamine	DB04633	Exper	1.098	1.946	-4.874	I	0.008
Cetyl alcohol	DB09494	Approved	1.134	2.303	-3.833	I	0.021
Undecanal	DB04093	Exper	1.413	1.609	-5.855	I	0.003
2-{2-[2-2-(Methoxy-Ethoxy)-Ethoxy]-Ethoxy}-Ethanol	DB04332	Exper	1.578	1.946	-4.874	I	0.008
Trolamine salicylate	DB11079	Approved	1.736	1.099	-7.345	I	0.001
MDL72527	DB04188	Exper	1.843	1.946	-4.874	I	0.008
Nonan-1-Ol	DB03143	Exper	1.980	1.099	-7.345	I	0.001
Hydrogen peroxide	DB11091	Approved; Vet	2.196	0.000	-10.550	I	0.000
Triethylene glycol	DB02327	Exper	2.428	1.099	-7.345	I	0.001

Exper.Exper.; Invest. Investigational; vet: vet approved;A:active;I:Inactive.

Table S2. DF_{Class_6LU7} external set: compounds identification, 6LU7 Mpro docking score, descriptors value, classification and probability of being classified as active.

Compound	Drugbank id.	Drugbank category	Docking score (6LU7)	MPC08	DF_{Class_6LU7}	Class.	P.A.
**Active group**							
Coenzyme F420	DB03913	Exper.	-10.613	5.521	5.556	A	0.996
Bleomycin	DB00290	Approved; Invest.	-10.150	6.033	7.049	A	0.999
Coa-S-Acetyl 5-Bromotryptamine	DB03341	Exper.	-10.141	4.905	3.759	A	0.977
Carbobenzoxy-Pro-Lys-Phe-Y(Po2)-Ala-Pro-Ome	DB01989	Exper.	-10.073	5.384	5.157	A	0.994
Icatibant	DB06196	Approved; Invest.	-9.686	5.666	5.979	A	0.997
BV2	DB02574	Exper.	-9.526	5.617	5.834	A	0.997
Glutathionylspermidine Disulfide	DB02553	Exper.	-9.501	5.724	6.146	A	0.998
Angiotensinamide	DB13517	Exper.	-9.137	5.464	5.388	A	0.995
CBZ-LEU-LEU-TYR-CH2F	DB04653	Exper.	-9.039	5.635	5.887	A	0.997
8-epi-Cyanocobalamin	DB14092	Exper.	-8.959	5.464	5.388	A	0.995
9-hydroxy-6-(3-hydroxypropyl)-4-(2-methoxyphenyl)pyrrolo[3,4-c]carbazole-1,3(2h,6h)-dione	DB07006	Exper.	-8.908	5.236	4.725	A	0.991
Enalkiren	DB03395	Exper.	-8.824	4.754	3.316	A	0.965
Suramin	DB04786	Invest.	-8.687	4.205	1.715	A	0.848
N-{1-[5-(1-Carbamoyl-2-Mercapto-Ethylcarbamoyl)-Pentylcarbamoyl]-2-[4-(Difluoro-Phosphono-Methyl)-Phenyl]-Ethyl}-3-{2-[4-(Difluoro-Phosphono-Methyl)-Phenyl]-Acetylamino}-Succinamic Acid	DB03557	Exper.	-8.617	5.501	5.497	A	0.996
2-(Carboxymethoxy)-5-[(2s)-2-({(2s)-2-[(3-Carboxypropanoyl)Amino] -3-Phenylpropanoyl}Amino)-3-Oxo-3-(Pentylamino)Propyl]Benzoic Acid	DB04525	Exper.	-8.552	4.804	3.463	A	0.970
Delparantag	DB12955	Invest.	-8.481	5.293	4.891	A	0.993
Inhibitor Msa367	DB03803	Exper.	-8.216	5.771	6.285	A	0.998
Fosaprepitant	DB06717	Approved	-8.187	5.787	6.330	A	0.998
Isavuconazonium	DB06636	Approved; Invest.	-8.186	5.602	5.791	A	0.997
Sincalide	DB09142	Approved	-8.181	5.384	5.157	A	0.994
4-Benzoylamino-4-{1-{1-Carbamoyl-2-[4-(Difluoro-Phosphono-Methyl)-Phenyl]-Ethylcarbamoyl}-2-[4-(Difluoro-Phosphono-Methyl)-Phenyl]-	DB03483	Exper.	-8.159	5.347	5.048	A	0.994

Ethylcarbamoyl}-Butyric Acid							
LFA703	DB03932	Exper.	-8.124	5.700	6.078	A	0.998
Difelikefalin	DB11938	Invest.	-8.105	5.609	5.813	A	0.997
Iomeprol	DB11705	Approved; Invest.	-8.060	5.541	5.614	A	0.996
4-(N,N-Dimethylamino)Cinnamoyl-Coa	DB04117	Exper.	-8.055	6.685	8.949	A	1.000
Rotigaptide	DB13067	Invest.	-8.031	5.464	5.388	A	0.995
**Inactive Group**							
Glycine	DB00145	Approved; Nutraceutical ; Vet	-1.960	0.000	-10.550	I	0.000
Aluminium glycinate	DB13626	Exper.	-1.956	0.000	-10.550	I	0.000
Argininosuccinate	DB02267	Exper.	-1.864	2.639	-2.852	I	0.055
1-Monohexanoyl-2-Hydroxy-Sn-Glycero-3-Phosphate	DB04199	Exper.	-1.848	2.485	-3.302	I	0.036
(5r)-5-Amino-6-Hydroxyhexylcarbamic Acid	DB02437	Exper.	-1.764	1.792	-5.323	I	0.005
Hypophosphite	DB04053	Exper.	-1.739	0.000	-10.550	I	0.000
Nitroarginine	DB04223	Exper;. Invest.	-1.691	2.197	-4.141	I	0.016
3-(1-Aminoethyl)Nonanedioic Acid	DB02941	Exper.	-1.637	2.303	-3.833	I	0.021
N-omega-nitro-L-arginine methyl ester	DB12750	Invest.	-1.523	2.303	-3.833	I	0.021
1-(4-hexylphenyl)prop-2-en-1-one	DB08085	Exper.	-1.409	2.079	-4.484	I	0.011
1-decane-sulfonic-acid	DB06893	Exper.	-1.332	1.946	-4.874	I	0.008
OTX-008	DB13123	Invest.	-1.311	5.855	6.529	A	0.999
Ethambutol	DB00330	Approved	-1.241	2.197	-4.141	I	0.016
Linoleic acid	DB14104	Approved; Exper.	-1.226	2.565	-3.068	I	0.044
Propatyl nitrate	DB13255	Exper.; Invest.	-1.221	2.565	-3.068	I	0.044
Lauryl Dimethylamine-N-Oxide	DB04147	Exper.	-1.185	2.197	-4.141	I	0.016
Stearic acid	DB03193	Approved; Exper.	-1.174	2.565	-3.068	I	0.044
methoxyundecylphosphinic acid	DB08222	Exper.	-1.085	2.197	-4.141	I	0.016
Decyl(dimethyl)phosphine oxide	DB07641	Exper.	-1.053	1.946	-4.874	I	0.008
4r-Fluoro-N6-Ethanimidoyl-L-Lysine	DB01835	Exper.	-0.926	1.946	-4.874	I	0.008
S-nonyl-cysteine	DB07849	Exper.	-0.898	2.197	-4.141	I	0.016
4-Oxosebacic Acid	DB02260	Exper.	-0.810	2.197	-4.141	I	0.016
Undecyl-Phosphinic Acid Butyl Ester	DB02457	Exper.	-0.770	2.398	-3.555	I	0.028
Octyldodecanol	DB14134	Exper.	-0.461	2.773	-2.462	I	0.079
2-octyl cyanoacrylate	DB12040	Invest.	-0.429	1.946	-4.874	I	0.008

Diethylhomospermine	DB13011	Invest.	-0.337	2.565	-3.068	I	0.044
11-mercaptoundecanoic acid	DB08171	Exper.	-0.318	1.946	-4.874	I	0.008
2-(2-{2-[2-(2-Methoxy-Ethoxy)-Ethoxy]-Ethoxy}-Ethoxy)-Ethanol	DB02042	Exper.	-0.253	2.303	-3.833	I	0.021
Palmidrol	DB14043	Exper.; Nutraceutical	-0.168	2.639	-2.852	I	0.055
undecan-2-one	DB08688	Exper.	-0.039	1.609	-5.855	I	0.003
N-Tridecanoic Acid	DB02448	Exper.	0.042	2.079	-4.484	I	0.011
Myristic acid	DB08231	Exper.	0.072	2.197	-4.141	I	0.016
Capric acid	DB03600	Exper.	0.106	1.609	-5.855	I	0.003
Diethylnorspermine	DB06445	Invest.	0.113	2.303	-3.833	I	0.021
N-Octyl-2-Hydroxyethyl Sulfoxide	DB02415	Exper.	0.151	1.792	-5.323	I	0.005
Tilarginine	DB11815	Approved; Invest.	0.199	1.946	-4.874	I	0.008
1-ethoxy-2-(2-ethoxyethoxy)ethane	DB08357	Exper.	0.676	1.386	-6.506	I	0.001
hexadecanal	DB03381	Exper.	0.752	2.303	-3.833	I	0.021
decyl formate	DB07650	Exper.	0.902	1.792	-5.323	I	0.005
1-(hydroxymethyleneamino)-8-hydroxy-octane	DB07897	Exper.	1.075	1.609	-5.855	I	0.003
Trolamine	DB13747	Approved	1.747	0.000	-10.550	I	0.000
Coumermycin A1	DB13912	Exper.	2.909	6.211	7.566	A	0.999

Exper. Exper. ; Invest. Investigational; vet: vet approved

Table S3. ANN_{Class_6LU7} training set: compounds identification, 6LU7 Mpro docking score, descriptors value, classification and confidence level associated.

Compound	Docking score (6LU7)	MPC08	ANN_{Class_6LU7} Class.	Conf. levels
**Active group**				
Gonadorelin	-11.695	5.684	A	0.873
Ornipressin	-11.367	5.609	A	0.873
Felypressin	-10.94	4.956	A	0.872
Carbetocin	-10.381	5.380	A	0.873
(E)-(4S,6S)-6-((S)-2-{(S)-2-[(furan-2-carbonyl)-amino]-3-methyl-butyrylamino}-3-methyl-butyrylamino)-8-methyl-5-oxo-4-((r)-2-oxo-pyrrolidin-3-ylmethyl)-non-2-enoic acid ethyl ester	-10.222	5.897	A	0.873
Iotrolan	-10.176	4.605	A	0.869
Lypressin	-10.107	5.513	A	0.873
Triptorelin	-10.045	5.945	A	0.873
Lanreotide	-9.973	4.956	A	0.872
Birinapant	-9.893	6.084	A	0.873
Indium In-111 pentetreotide	-9.877	6.116	A	0.873
Ipamorelin	-9.873	4.754	A	0.870
UK-432097	-9.697	5.756	A	0.873
Pentagastrin	-9.648	4.984	A	0.872
Terlipressin	-9.511	6.192	A	0.873
Succinamide-CoA	-9.472	5.493	A	0.873
Iopamidol	-9.397	5.464	A	0.873
BV1	-9.387	5.549	A	0.873
Labradimil	-9.377	5.371	A	0.873
Etelcalcetide	-9.353	4.942	A	0.872
LY231514 Tetra Glu	-9.318	4.812	A	0.871
talactoferrin alpha	-9.217	5.333	A	0.873
8-Demethyl-8-Dimethylamino-Flavin-Adenine-Dinucleotide	-9.206	4.533	A	0.867
N-(Sulfanylacetyl)Tyrosylprolylmethioninamide	-9.162	3.970	A	0.819
Colistin	-9.125	5.357	A	0.873
4'-nitrophenyl-3i-thiolaminaritrioside	-9.014	5.858	A	0.873
Hydroxyethyl cellulose	-9.014	5.684	A	0.873
S-(2-Oxo)Pentadecylcoa	-9	5.069	A	0.872
4-Hydroxyphenacyl Coenzyme A	-8.999	5.416	A	0.873
Coa-S-Acetyl Tryptamine	-8.927	5.541	A	0.873
Vapreotide	-8.904	5.762	A	0.873
Adrabetadex	-8.894	6.436	A	0.873
Thymopentin	-8.889	4.554	A	0.868
Saquinavir	-8.844	5.403	A	0.873
3-Thiaoctanoyl-Coenzyme A	-8.745	4.007	A	0.827

4-methyl-pentanoic acid {1-[4-guanidino-1-(thiazole-2-carbonyl)-butylcarbamoyl]-2-methyl-propyl}-amide	-8.745	5.580	A	0.873
Sinapoyl Coenzyme A	-8.736	4.984	A	0.872
P1-(5'-Adenosyl)P5-(5'-Thymidyl)Pentaphosphate	-8.726	4.500	A	0.867
Dotatate	-8.708	5.509	A	0.873
3-[3-(2,3-Dihydroxy-Propylamino)-Phenyl]-4-(5-Fluoro-1-Methyl-1h-Indol-3-Yl)-Pyrrole-2,5-Dione	-8.697	5.063	A	0.872
Iobitridol	-8.697	5.112	A	0.872
Angiotensin II	-8.674	5.485	A	0.873
Flavin adenine dinucleotide	-8.539	5.476	A	0.873
Iodixanol	-8.538	5.017	A	0.872
Sufugolix	-8.501	4.234	A	0.855
Elamipretide	-8.476	5.768	A	0.873
Flavin-N7 Protonated-Adenine Dinucleotide	-8.446	5.976	A	0.873
Alatrofloxacin	-8.428	5.704	A	0.873
Methylmalonyl-Coenzyme A	-8.428	5.004	A	0.872
Coa-S-Trimethylene-Acetyl-Tryptamine	-8.422	5.513	A	0.873
Trifluoroacetonyl Coenzyme A	-8.388	5.624	A	0.873
Je-2147, Ag1776, Kni-764	-8.329	5.198	A	0.872
Giripladib	-8.314	4.779	A	0.871
Efrotomycin	-8.288	4.615	A	0.869
3-[1-(3-aminopropyl)-1h-indol-3-yl]-4-(1h-indol-3-yl)-1h-pyrrole-2,5-dione	-8.259	5.347	A	0.873
Fosifloxuridine nafalbenamide	-8.25	5.268	A	0.873
HM-30181	-8.238	4.466	A	0.866
2-Hydroxy-5-({1-[(4-Methylphenoxy)Methyl]-3-Oxoprop-1-Enyl}Amino)-L-Tyrosine	-8.232	4.277	A	0.858
Caspofungin	-8.208	4.927	A	0.872
Benzoyl-Arginine-Alanine-Methyl Ketone	-8.2	3.761	A	0.735
Inhibitor Bea388	-8.191	5.493	A	0.873
3-[(5s)-1-Acetyl-3-(2-Chlorophenyl)-4,5-Dihydro-1h-Pyrazol-5-Yl]Phenol	-8.186	5.380	A	0.873
Cefbuperazone	-8.171	4.500	A	0.867
Telinavir	-8.164	5.961	A	0.873
Nicotinamide-Adenine-Dinucleotide-5-Hydroxy-4-Oxonorvaline	-8.154	5.609	A	0.873
Cangrelor	-8.151	5.557	A	0.873
Polymyxin B	-8.149	5.489	A	0.873
N-(4-carbamimidoylbenzyl)-1-(4-methylpentanoyl)-L-prolinamide	-8.129	5.338	A	0.873
NUC-1031	-8.118	4.949	A	0.872
Larazotide	-8.117	5.533	A	0.873
Inarigivir soproxil	-8.105	5.677	A	0.873
Cephalosporin Analog	-8.077	5.308	A	0.873
FR236913	-8.056	5.176	A	0.872
TOP-1288	-8.054	4.883	A	0.871

Ioforminol	-8.05	4.762	A	0.871
Dirlotapide	-8.034	4.898	A	0.872
BMS-986094	-8.021	6.033	A	0.873
Iopentol	-8.016	4.543	A	0.868
Carfilzomib	-8.009	5.056	A	0.872
4-Oxo-Nicotinamide-Adenine Dinucleotide Phosphate	-8.002	5.919	A	0.873
**Inactive group**				
Agmatine	-2,000	0.000	I	1.000
1-acetyl-2-lyso-sn-glycero-3-phosphoethanolamine	-1.961	2.398	I	1.000
Glycerin	-1.961	0.000	I	1.000
Magnesium glycinate	-1.96	0.000	I	1.000
Zinc glycinate	-1.96	0.000	I	1.000
Aluminum zirconium octachlorohydrex gly	-1.956	0.000	I	1.000
Dihydroxyacetone	-1.956	0.000	I	1.000
Ferrous glycine sulfate	-1.956	0.000	I	1.000
(S)-2-Amino-4-[(2s,3r)-2,3,5-Trihydroxy-4-Oxo-Pentyl]Mercapto-Butyric Acid	-1.908	2.485	I	1.000
1,4-Butanediol	-1.859	0.000	I	1.000
Amyl Nitrite	-1.822	0.000	I	1.000
3-Oxo-Pentadecanoic Acid	-1.815	2.398	I	1.000
4-Hydroxybutan-1-Aminium	-1.809	0.000	I	1.000
NB-001	-1.783	3.807	A	0.761
L-cystein-s-1-(iminomethyl)-l-ornithine	-1.733	2.485	I	1.000
L-Alpha-Glycerophosphorylserine	-1.729	2.079	I	1.000
2-Hydroxyethyl Disulfide	-1.698	0.000	I	1.000
Ricinoleic Acid	-1.612	2.639	I	1.000
cis-Vaccenic acid	-1.61	2.565	I	1.000
Tylosin	-1.587	5.568	A	0.873
Dexpanthenol	-1.578	1.609	I	1.000
Nz-(1-Carboxyethyl)-Lysine	-1.566	2.197	I	1.000
Troleandomycin	-1.566	5.513	A	0.873
N-(Phosphonoacetyl)-L-Ornithine	-1.542	2.303	I	1.000
Sevelamer	-1.522	0.000	I	1.000
3,7,11,15-tetramethyl-hexadecan-1-ol	-1.515	2.708	I	1.000
Nz-(Dicarboxymethyl)Lysine	-1.508	2.398	I	1.000
L-Alpha-Glycerophosphorylethanolamine	-1.502	1.386	I	1.000
1,3-Propandiol	-1.482	0.000	I	1.000
N-Alpha-L-Acetyl-Arginine	-1.475	2.197	I	1.000
Methylethylamine	-1.437	0.000	I	1.000
(9Z,11E,13S)-13-hydroxyoctadeca-9,11-dienoic acid	-1.41	2.639	I	1.000
(3R)-3-hydroxydodecanoic acid	-1.409	2.944	I	1.000
N-Butyl-N'-Hydroxyguanidine	-1.371	0.000	I	1.000
2-Decenoyl N-Acetyl Cysteamine	-1.357	2.398	I	1.000

4-amino-n-[(2-sulfanylethyl)carbamoyl]benzenesulfonamide	-1.335	2.833	I	1.000
Cimetidine	-1.306	2.773	I	1.000
undecylamine-n,n-dimethyl-n-oxide	-1.302	2.079	I	1.000
MF268	-1.288	2.944	I	1.000
Triethoxycaprylylsilane	-1.283	2.398	I	1.000
Ethyl Isocyanide	-1.203	0.000	I	1.000
Alpha-linolenic acid	-1.198	2.565	I	1.000
Sodium lauryl sulfate	-1.178	2.303	I	1.000
Dodecyl sulfate	-1.174	2.197	I	1.000
Magnesium stearate	-1.174	3.219	I	0.988
Guanidine-3-Propanol	-1.153	0.000	I	1.000
Dolastatin 10	-1.137	5.030	A	0.872
(10E,12Z)-octadecadienoic acid	-1.114	2.565	I	1.000
S-(D-Carboxybutyl)-L-Homocysteine	-1.108	2.079	I	1.000
N2-(Carboxyethyl)-L-Arginine	-1.104	2.398	I	1.000
diethyl propane-1,3-diylbiscarbamate	-1.072	2.197	I	1.000
GW-274150	-1.06	1.946	I	1.000
Etoglucid	-1.057	2.708	I	1.000
Palmitoleic Acid	-1.019	2.398	I	1.000
Odalasvir	-1.013	6.526	A	0.873
3-Hydroxy-Myristic Acid	-1.002	2.303	I	1.000
Nitrosoethane	-0.974	0.000	I	1.000
Elaidoylamide	-0.953	2.565	I	1.000
Bromo-Dodecanol	-0.947	1.946	I	1.000
Ethylhexylglycerin	-0.914	1.792	I	1.000
Rifampicin	-0.888	5.958	A	0.873
1-Guanidinium-7-Aminoheptane	-0.881	1.609	I	1.000
Ethanolamine oleate	-0.866	2.565	I	1.000
Ethanol	-0.776	0.000	I	1.000
C31G	-0.753	2.996	I	1.000
n-dodecyl-n,n-dimethylglycinate	-0.753	2.485	I	1.000
5-N-Allyl-Arginine	-0.752	2.197	I	1.000
Hexaminolevulinate	-0.718	2.079	I	1.000
Cetrimonium	-0.607	2.565	I	1.000
Dibutylsuccinate	-0.599	2.197	I	1.000
2-[3-(2-Hydroxy-1,1-Dihydroxymethyl-Ethylamino)-Propylamino]-2-Hydroxymethyl-Propane-1,3-Diol	-0.567	2.773	I	1.000
Tiadenol	-0.524	2.398	I	1.000
N5-(1-Imino-3-Butenyl)-L-Ornithine	-0.514	2.079	I	1.000
3,6,9,12,15-Pentaoxaheptadecane	-0.513	2.303	I	1.000
N-Omega-Propyl-L-Arginine	-0.511	2.197	I	1.000
Dioctyldimonium	-0.501	2.639	I	1.000
Decamethonium	-0.471	2.398	I	1.000

11-[(mercaptocarbonyl)oxy]undecanoic acid	-0.459	2.398	I	1.000
N-Omega-Hydroxy-L-Arginine	-0.453	2.303	I	1.000
Dodecane-Trimethylamine	-0.414	1.946	I	1.000
Hydroxybutyloxide	-0.41	2.197	I	1.000
Tris(Hydroxyethyl)Aminomethane	-0.395	1.609	I	1.000
Lauroyl chloride	-0.364	0.000	I	1.000
12-Hydroxydodecanoic Acid	-0.358	1.946	I	1.000
Diethylhomospermine	-0.348	2.079	I	1.000
Oleic Acid	-0.337	1.609	I	1.000
Silanol	-0.319	2.565	I	1.000
N3, N4-Dimethylarginine	-0.311	0.000	I	1.000
Pentaglyme	-0.295	2.197	I	1.000
Ocrylate	-0.221	2.398	I	1.000
Isopropyl myristate	-0.198	2.079	I	1.000
Monoctanoin	-0.188	2.485	I	1.000
3,6,9,12,15,18-hexaoxaicosane	-0.151	2.079	I	1.000
Vinyl ether	-0.112	2.565	I	1.000
NCX 701	-0.108	0.000	I	1.000
Trolnitrate	-0.094	3.367	I	0.867
Methyl Nonanoate (Ester)	-0.094	2.773	I	1.000
Palmitic Acid	-0.084	1.609	I	1.000
sebacic acid	-0.081	2.398	I	1.000
Azelaic acid	-0.07	1.946	I	1.000
Bombykol	-0.064	1.792	I	1.000
Nitrous acid	-0.004	2.303	I	1.000
L-Homoarginine	0.022	0.000	I	1.000
O-Decyl Hydrogen Thiocarbonate	0.04	1.946	I	1.000
Lauric acid	0.106	1.946	I	1.000
Spermine	0.148	1.946	I	1.000
Quaternium-24	0.161	1.946	I	1.000
Rifapentine	0.164	2.773	I	1.000
Tetraglyme	0.328	6.019	A	0.873
3,6,9,12,15-pentaoxaheptadecan-1-ol	0.353	2.079	I	1.000
5-(2-hydroxyethyl)nonane-1,9-diol	0.541	1.792	I	1.000
Triglyme	0.62	1.609	I	1.000
decane-1-thiol	0.704	1.386	I	1.000
tetrabutylammonium ion	0.711	1.946	I	1.000
undecylenic acid	0.719	1.792	I	1.000
1-dodecanol	0.953	1.792	I	1.000
bis(hexamethylene)triamine	0.953	2.079	I	1.000
N-ethyl-N-[3-(propylamino)propyl]propane-1,3-diamine	1.098	1.946	I	1.000
Cetyl alcohol	1.134	2.303	I	1.000
Undecanal	1.413	1.609	I	1.000

2-{2-[2-2-(Methoxy-Ethoxy)-Ethoxy]-Ethoxy}-Ethanol	1.578	1.946	I	1.000
Trolamine salicylate	1.736	1.099	I	1.000
MDL72527	1.843	1.946	I	1.000
Nonan-1-Ol	1.98	1.099	I	1.000
Hydrogen peroxide	2.196	0.000	I	1.000
Triethylene glycol	2.428	1.099	I	1.000

Table S4. ANN_{Class_6LU7} external set: compounds identification, 6LU7 Mpro docking score, descriptors value, classification and confidence level associated.

Compound	Docking score (6LU7)	MPC08	ANN_{Class_6LU7} Pred	Conf. levels
**Test Active group**				
Coenzyme F420	-10.613	5.521	A	0.873
Bleomycin	-10.150	6.033	A	0.873
Coa-S-Acetyl 5-Bromotryptamine	-10.141	4.905	A	0.872
Carbobenzoxy-Pro-Lys-Phe-Y(Po2)-Ala-Pro-Ome	-10.073	5.384	A	0.873
Icatibant	-9.686	5.666	A	0.873
BV2	-9.526	5.617	A	0.873
Glutathionylspermidine Disulfide	-9.501	5.724	A	0.873
Angiotensinamide	-9.137	5.464	A	0.873
CBZ-LEU-LEU-TYR-CH2F	-9.039	5.635	A	0.873
8-epi-Cyanocobalamin	-8.959	5.464	A	0.873
9-hydroxy-6-(3-hydroxypropyl)-4-(2-methoxyphenyl)pyrrolo[3,4-c]carbazole-1,3(2h,6h)-dione	-8.908	5.236	A	0.873
Enalkiren	-8.824	4.754	A	0.870
Suramin	-8.687	4.205	A	0.853
N-{1-[5-(1-Carbamoyl-2-Mercapto-Ethylcarbamoyl)-Pentylcarbamoyl]-2-[4-(Difluoro-Phosphono-Methyl)-Phenyl]-Ethyl}-3-{2-[4-(Difluoro-Phosphono-Methyl)-Phenyl]-Acetylamino}-Succinamic Acid	-8.617	5.501	A	0.873
2-(Carboxymethoxy)-5-[(2s)-2-({(2s)-2-[(3-Carboxypropanoyl)Amino] -3-Phenylpropanoyl}Amino)-3-Oxo-3-(Pentylamino)Propyl]Benzoic Acid	-8.552	4.804	A	0.871
Delparantag	-8.481	5.293	A	0.873
Inhibitor Msa367	-8.216	5.771	A	0.873
Fosaprepitant	-8.187	5.787	A	0.873
Isavuconazonium	-8.186	5.602	A	0.873
Sincalide	-8.181	5.384	A	0.873
4-Benzoylamino-4-{1-{1-Carbamoyl-2-[4-(Difluoro-Phosphono-Methyl)-Phenyl]-Ethylcarbamoyl}-2-[4-(Difluoro-Phosphono-Methyl)-Phenyl]-Ethylcarbamoyl}-Butyric Acid	-8.159	5.347	A	0.873
LFA703	-8.124	5.700	A	0.873
Difelikefalin	-8.105	5.609	A	0.873
Iomeprol	-8.06	5.541	A	0.873
4-(N,N-Dimethylamino)Cinnamoyl-Coa	-8.055	6.685	A	0.873
Rotigaptide	-8.031	5.464	A	0.873
**Test inactive group**				
Glycine	-1.960	0.000	I	1.000
1-Monohexanoyl-2-Hydroxy-Sn-Glycero-3-Phosphate	-1.848	2.485	I	1.000

Aluminium glycinate	-1.822	0.000	I	1.000
(5r)-5-Amino-6-Hydroxyhexylcarbamic Acid	-1.764	1.792	I	1.000
Hypophosphite	-1.739	0.000	I	1.000
Nitroarginine	-1.691	2.197	I	1.000
3-(1-Aminoethyl)Nonanedioic Acid	-1.637	2.303	I	1.000
N-omega-nitro-L-arginine methyl ester	-1.523	2.303	I	1.000
1-decane-sulfonic-acid	-1.332	1.946	I	1.000
Otx-008	-1.311	5.855	A	0.873
Linoleic acid	-1.226	2.565	I	1.000
Propatyl nitrate	-1.221	2.565	I	1.000
Lauryl dimethylamine-n-oxide	-1.185	2.197	I	1.000
Stearic acid	-1.174	2.565	I	1.000
Methoxyundecylphosphinic acid	-1.085	2.197	I	1.000
Diethylnorspermine	-1.072	2.303	I	1.000
4r-Fluoro-N6-Ethanimidoyl-L-Lysine	-0.926	1.946	I	1.000
S-nonyl-cysteine	-0.898	2.197	I	1.000
4-Oxosebacic Acid	-0.810	2.197	I	1.000
Ethambutol	-0.776	2.197	I	1.000
Undecyl-Phosphinic Acid Butyl Ester	-0.77	2.398	I	1.000
Octyldodecanol	-0.461	2.773	I	1.000
2-octyl cyanoacrylate	-0.429	1.946	I	1.000
11-mercaptoundecanoic acid	-0.318	1.946	I	1.000
2-(2-{2-[2-(2-Methoxy-Ethoxy)-Ethoxy]-Ethoxy}-Ethoxy)-Ethanol	-0.253	2.303	I	1.000
Palmidrol	-0.168	2.639	I	1.000
undecan-2-one	-0.039	1.609	I	1.000
N-Tridecanoic Acid	0.042	2.079	I	1.000
Myristic acid	0.072	2.197	I	1.000
N-Octyl-2-Hydroxyethyl Sulfoxide	0.151	1.792	I	1.000
Tilarginine	0.199	1.946	I	1.000
1-ethoxy-2-(2-ethoxyethoxy)ethane	0.676	1.386	I	1.000
Coumermycin A1	0.704	6.211	A	0.873
Hexadecanal	0.752	2.303	I	1.000
Argininosuccinate	0.953	2.639	I	1.000
1-(hydroxymethyleneamino)-8-hydroxy-octane	1.075	1.609	I	1.000
Capric acid	1.134	1.609	I	1.000
Decyl formate	1.479	1.946	I	1.000
Trolamine	1.747	0.000	I	1.000

Table S5. MLRA$_{reg_6LU7}$ training set: compounds identification, 6LU7 Mpro docking score, descriptors value and 6LU7 Mpro docking score predicted.

**Compound**	**Docking score (6LU7)**	**SpDiam_EA(bo)**	**Eig09_EA(bo)**	**nRNR2**	**N-068**	**CATS2D_05_LL**	**nLevel1**	**Docking score (6LU7) Predicted**
**Active group**								
Gonadorelin	-11.695	7.474	2.998	0	0	6	3	-9.576
Ornipressin	-11.367	7.581	2.883	0	0	0	3	-10.178
Felypressin	-10.940	5.292	2.710	0	0	5	3	-7.706
Carbetocin	-10.381	7.617	2.153	0	0	0	3	-8.918
(E)-(4s,6s)-6-((s)-2-{(s)-2-[(furan-2-carbonyl)-amino]-3-methyl-butyrylamino}-3-methyl-butyrylamino)-8-methyl-5-oxo-4-((r)-2-oxo-pyrrolidin-3-ylmethyl)-non-2-enoic acid ethyl ester	-10.222	7.474	3.069	4	4	3	3	-7.411
Iotrolan	-10.176	7.505	1.360	0	0	0	3	-7.447
Lypressin	-10.107	7.524	2.376	0	0	28	3	-5.832
Triptorelin	-10.045	7.474	3.139	0	0	9	3	-9.458
Lanreotide	-9.973	7.084	2.077	0	0	3	3	-8.056
Birinapant	-9.893	7.714	2.705	0	0	16	3	-8.003
Indium In-111 pentetreotide	-9.877	6.848	3.050	0	0	8	3	-8.998
Ipamorelin	-9.873	7.500	2.099	0	0	4	3	-8.255
UK-432097	-9.697	7.625	2.676	0	0	12	3	-8.381
Pentagastrin	-9.648	6.904	2.093	0	0	1	3	-8.205
Terlipressin	-9.511	7.992	3.971	0	0	28	0	-7.619
Succinamide-Coa	-9.472	7.474	3.012	0	0	6	3	-9.600
Iopamidol	-9.397	7.498	2.393	0	0	0	3	-9.260
BV1	-9.387	7.618	2.842	0	0	3	3.2	-9.855
Labradimil	-9.377	7.133	2.815	0	0	8	3.3	-8.926
Etelcalcetide	-9.353	6.645	2.506	0	0	5	3	-8.268
LY231514 Tetra Glu	-9.318	5.430	2.642	0	0	5	3	-7.682
Talactoferrin alpha	-9.217	7.581	2.911	0	0	1	3	-10.105
8-Demethyl-8-Dimethylamino-Flavin-Adenine-Dinucleotide	-9.206	6.982	1.200	0	0	8	3	-5.832
N-(Sulfanylacetyl)Tyrosylprolylmethioninamide	-9.162	6.756	0.953	0	0	3	3	-5.854
Colistin	-9.125	7.162	2.775	0	0	3	3	-9.336

4'-nitrophenyl-3i-thiolaminaritriosi de	-9.014	7.581	2.914	0	0	0	3	-10.233
Hydroxyethyl cellulose	-9.014	7.474	2.998	0	0	6	3	-9.576
S-(2-Oxo)Pentadecylc oa	-9.000	7.474	2.683	0	0	5	3	-9.143
4-Hydroxyphenacy l Coenzyme A	-8.999	7.581	3.002	0	0	4	3	-9.899
Coa-S-Acetyl Tryptamine	-8.927	7.581	3.006	0	0	5	3	-9.784
Vapreotide	-8.904	7.474	3.142	0	0	5	3	-9.952
Adrabetadex	-8.894	5.264	2.571	0	0	0	3	-8.054
Thymopentin	-8.889	6.625	2.654	0	0	1	3	-9.004
Saquinavir	-8.844	6.477	2.941	0	0	9	3	-8.432
3-Thiaoctanoyl-Coenzyme A	-8.745	6.367	1.310	0	0	5	3	-5.974
4-methyl-pentanoic acid {1-[4-guanidino-1-(thiazole-2-carbonyl)-butylcarbamoyl]-2-methyl-propyl}-amide	-8.745	7.581	2.743	0	0	0	3	-9.932
Sinapoyl Coenzyme A	-8.736	6.630	2.629	0	0	4	3	-8.596
P1-(5'-Adenosyl)P5-(5'-Thymidyl)Pentap hosphate	-8.726	6.629	1.500	0	0	3	3	-6.731
Dotatate	-8.708	7.607	2.890	0	0	16	3	-8.257
3-[3-(2,3-Dihydroxy-Propylamino)-Phenyl]-4-(5-Fluoro-1-Methyl-1h-Indol-3-Yl)-Pyrrole-2,5-Dione	-8.697	6.930	2.140	0	0	0	3	-8.428
Iobitridol	-8.697	7.231	2.803	0	0	5	3	-9.189
Angiotensin II	-8.674	6.626	2.989	0	0	8	3	-8.739
Flavin adenine dinucleotide	-8.539	6.486	2.880	0	0	4	3	-8.940
Iodixanol	-8.538	6.516	2.484	0	0	0	3	-8.753
Sufugolix	-8.501	6.756	1.493	0	0	1	3	-7.048
Elamipretide	-8.476	6.845	2.877	0	0	19	3.05	-7.371
Flavin-N7 Protonated-Adenine Dinucleotide	-8.446	7.323	2.862	0	0	4	3	-9.477
Alatrofloxacin	-8.428	7.272	2.461	0	0	10	3	-8.006
Methylmalonyl-Coenzyme A	-8.428	7.214	2.749	0	0	14	3	-7.985
Coa-S-Trimethylene-	-8.422	7.581	2.994	0	0	4	3	-9.886

Acetyl-Tryptamine								
Trifluoroacetonyl Coenzyme A	-8.388	6.626	2.926	0	0	4	3	-9.117
Je-2147, Ag1776, Kni-764	-8.329	7.336	2.784	0	0	9	3	-8.739
Giripladib	-8.314	7.504	2.046	0	0	1	3	-8.530
Efrotomycin	-8.288	6.329	2.120	0	0	9	3	-6.885
3-[1-(3-aminopropyl)-1h-indol-3-yl]-4-(1h-indol-3-yl)-1h-pyrrole-2,5-dione	-8.259	7.581	2.825	0	0	4	3	-9.588
Fosifloxuridine nafalbenamide	-8.250	7.068	2.322	1	1	3	3	-7.812
HM-30181	-8.238	5.907	1.904	0	0	2	3	-7.072
2-Hydroxy-5-({1-[(4-Methylphenoxy) Methyl]-3-Oxoprop-1-Enyl}Amino)-L-Tyrosine	-8.232	7.056	1.456	0	0	4	3	-6.822
Caspofungin	-8.208	6.737	2.322	1	1	5	3.2	-7.432
Benzoyl-Arginine-Alanine-Methyl Ketone	-8.200	6.455	1.348	0	0	1	3	-6.589
Inhibitor Bea388	-8.191	7.579	2.324	0	0	0	3	-9.192
3-[(5s)-1-Acetyl-3-(2-Chlorophenyl)-4,5-Dihydro-1h-Pyrazol-5-Yl]Phenol	-8.186	7.581	3.001	0	0	4	3	-9.897
Cefbuperazone	-8.171	6.631	2.153	0	0	2	3	-8.004
Telinavir	-8.164	7.431	2.597	1	1	10	0	-6.351
Nicotinamide-Adenine-Dinucleotide-5-Hydroxy-4-Oxonorvaline	-8.154	7.581	2.883	0	0	0	3	-10.178
Cangrelor	-8.151	7.099	2.961	4	4	4	3	-6.844
Polymyxin B	-8.149	6.626	2.886	0	0	4	3	-9.046
N-(4-carbamimidoylbenzyl)-1-(4-methylpentanoyl)-L-prolinamide	-8.129	7.581	2.911	0	0	2	3	-9.983
NUC-1031	-8.118	7.231	2.807	0	0	3	3	-9.441
Larazotide	-8.117	6.640	2.901	0	0	5	3	-8.960
Inarigivir soproxil	-8.105	5.268	2.210	0	0	0	3	-7.420
Cephalosporin Analog	-8.077	6.732	2.110	0	0	0	3.25	-8.351
FR236913	-8.056	7.439	2.427	0	0	3	3	-8.913
TOP-1288	-8.054	7.352	2.427	0	0	3	3	-8.854

Ioforminol	-8.050	7.503	1.689	0	0	4	3	-7.537
Dirlotapide	-8.034	6.583	2.096	0	0	5	3	-7.505
BMS-986094	-8.021	7.161	3.115	0	0	3	3	-9.934
Iopentol	-8.016	7.507	1.239	0	0	0	3	-7.234
Carfilzomib	-8.009	6.950	2.160	0	0	5	3	-7.866
4-Oxo-Nicotinamide-Adenine Dinucleotide Phosphate	-8.002	8.038	2.085	0	0	15	0	-5.917
**Inactive group**								
Agmatine	-2.000	4.660	0.000	0	0	0	0	-1.780
1-acetyl-2-lyso-sn-glycero-3-phosphoethanolamine	-1.961	5.782	-1.000	0	0	0	3	-2.121
Glycerin	-1.961	3.921	0.000	0	0	0	0	-1.277
Magnesium glycinate	-1.960	4.435	0.000	0	0	0	0	-1.627
Zinc glycinate	-1.960	4.435	0.000	0	0	0	0	-1.627
Aluminum zirconium octachlorohydrex gly	-1.956	4.435	0.000	0	0	0	0	-1.627
Dihydroxyacetone	-1.956	4.583	0.000	0	0	0	0	-1.727
Ferrous glycine sulfate	-1.956	6.372	-1.372	0	0	0	0	-0.529
(S)-2-Amino-4-[(2s,3r)-2,3,5-Trihydroxy-4-Oxo-Pentyl]Mercapto-Butyric Acid	-1.908	5.143	-0.750	0	0	0	3	-2.126
1,4-Butanediol	-1.859	3.464	0.000	0	0	0	0	-0.967
Amyl Nitrite	-1.822	3.900	0.000	0	0	0	0	-1.263
3-Oxo-Pentadecanoic Acid	-1.815	4.992	-0.355	0	0	8	0	-0.404
4-Hydroxybutan-1-Aminium	-1.809	3.464	0.000	0	0	0	0	-0.967
NB-001	-1.783	7.566	0.421	0	0	0	0	-4.497
L-cystein-s-1-(iminomethyl)-l-ornithine	-1.733	5.007	-0.774	0	0	1	3	-1.869
L-Alpha-Glycerophosphorylserine	-1.729	5.784	-1.000	0	0	0	3	-2.122
2-Hydroxyethyl Disulfide	-1.698	3.696	0.000	0	0	0	0	-1.124
Ricinoleic Acid	-1.612	5.028	0.232	0	0	10	3	-2.556
cis-Vaccenic acid	-1.610	5.019	0.235	0	0	12	0	-0.974
Tylosin	-1.587	6.055	2.468	1	1	16	3.05	-5.815
Dexpanthenol	-1.578	5.420	-1.334	0	0	1	3	-1.165

Nz-(1-Carboxyethyl)-Lysine	-1.566	4.991	-1.262	0	0	1	3	-1.000
Troleandomycin	-1.566	5.787	2.601	1	1	17	3.05	-5.748
N-(Phosphonoacetyl)-L-Ornithine	-1.542	5.748	-1.000	0	0	0	3	-2.098
Sevelamer	-1.522	4.417	0.000	0	0	0	3	-2.952
3,7,11,15-tetramethyl-hexadecan-1-ol	-1.515	4.428	-0.090	0	0	16	3	-0.850
Nz-(Dicarboxymethyl)Lysine	-1.508	5.271	-1.095	0	0	0	3	-1.605
L-Alpha-Glycerophosphorylethanolamine	-1.502	5.729	-1.365	0	0	0	3	-1.443
1,3-Propandiol	-1.482	3.236	0.000	0	0	0	0	-0.812
N-Alpha-L-Acetyl-Arginine	-1.475	5.085	-1.316	0	0	1	3	-0.970
Methylethylamine	-1.437	2.828	0.000	0	0	0	0	-0.534
(9Z,11E,13S)-13-hydroxyoctadeca-9,11-dienoic acid	-1.410	5.270	0.420	0	0	10	3	-3.052
(3r)-3-hydroxydodecanoic acid	-1.409	6.779	-0.502	0	0	10	0	-1.117
N-Butyl-N'-Hydroxyguanidine	-1.371	4.743	0.000	0	0	0	0	-1.836
2-Decenoyl N-Acetyl Cysteamine	-1.357	5.386	-0.267	0	0	6	0	-1.071
4-amino-n-[(2-sulfanylethyl)carbamoyl]benzenesulfonamide	-1.335	7.704	-0.602	0	0	0	0	-2.789
Cimetidine	-1.306	6.444	-0.206	0	0	0	0	-2.630
Undecylamine-n,n-dimethyl-n-oxide	-1.302	5.052	-1.000	0	0	5	0	0.324
MF268	-1.288	4.769	-0.128	1	1	3	3	-1.937
Triethoxycaprylylsilane	-1.283	5.280	-0.445	0	0	5	0	-0.808
Ethyl Isocyanide	-1.203	4.000	0.000	0	0	0	0	-1.331
Alpha-Linolenic Acid	-1.198	5.193	0.251	0	0	12	0	-1.120
Sodium lauryl sulfate	-1.178	6.423	-0.594	0	0	6	0	-1.202
Dodecyl sulfate	-1.174	6.423	-0.594	0	0	6	0	-1.202
Magnesium stearate	-1.174	4.703	1.477	0	0	24	0	-1.480
Guanidine-3-Propanol	-1.153	4.642	0.000	0	0	0	0	-1.768
Dolastatin 10	-1.137	6.615	2.239	1	1	10	3	-6.503

(10E,12Z)-octadecadienoic acid	-1.114	5.254	0.242	0	0	12	0	-1.146
S-(D-Carboxybutyl)-L-Homocysteine	-1.108	4.989	-1.239	0	0	1	3	-1.040
N2-(Carboxyethyl)-L-Arginine	-1.104	5.032	-0.896	0	0	1	3	-1.673
Diethyl propane-1,3-diylbiscarbamate	-1.072	4.833	-0.737	0	0	0	0	-0.599
GW-274150	-1.060	4.989	-1.275	0	0	1	3	-0.975
Etoglucid	-1.057	4.659	0.000	0	0	0	3	-3.117
Palmitoleic Acid	-1.019	5.019	-0.147	0	0	10	0	-0.545
Odalasvir	-1.013	7.471	2.999	0	0	54	3	-3.720
3-Hydroxy-Myristic Acid	-1.002	4.780	-0.492	0	0	7	3	-1.481
Nitrosoethane	-0.974	3.464	0.000	0	0	0	0	-0.967
Elaidoylamide	-0.953	5.020	0.232	0	0	12	0	-0.969
Bromo-Dodecanol	-0.947	3.900	-0.868	0	0	6	0	0.996
Ethylhexylglycerin	-0.914	4.419	-1.128	0	0	2	3	-0.725
Rifampicin	-0.888	7.907	2.718	1	2	19	3.11	-0.130
1-Guanidinium-7-Aminoheptane	-0.881	4.166	-1.328	0	0	0	0	0.894
Ethanolamine oleate	-0.866	5.020	0.602	0	0	12	0	-1.619
Ethanol	-0.776	2.000	0.000	0	0	0	0	0.029
C31G	-0.753	5.243	1.428	0	0	12	0	-3.225
N-dodecyl-n,n-dimethylglycinate	-0.753	5.243	-0.348	0	0	6	0	-0.831
5-N-Allyl-Arginine	-0.752	5.420	-1.259	0	0	0	3	-1.419
Hexaminolevulinate	-0.718	4.911	-1.037	0	0	2	0	0.119
Cetrimonium	-0.607	5.073	0.070	0	0	10	0	-0.964
Dibutylsuccinate	-0.599	4.918	-0.482	0	0	4	0	-0.618
2-[3-(2-Hydroxy-1,1-Dihydroxymethyl-Ethylamino)-Propylamino]-2-Hydroxymethyl-Propane-1,3-Diol	-0.567	5.273	0.213	0	0	0	0	-2.571
Tiadenol	-0.524	3.939	0.000	0	0	5	0	-0.680
N5-(1-Imino-3-Butenyl)-L-Ornithine	-0.514	4.989	-1.268	0	0	2	3	-0.866
3,6,9,12,15-Pentaoxaheptadecane	-0.513	3.932	-0.185	0	0	0	0	-0.960

N-Omega-Propyl-L-Arginine	-0.511	4.989	-1.143	0	0	0	3	-1.330
Dioctyldimonium	-0.501	5.150	0.072	0	0	6	0	-1.509
3,6,9,12,15-pentaoxaheptadecan-1-ol	0.471	3.939	0.000	0	0	0	0	-1.290
Decamethonium	-0.459	5.066	-0.845	0	0	3	0	-0.203
11-[(mercaptocarbonyl)oxy]undecanoic acid	-0.453	4.710	-0.582	0	0	4	0	-0.301
N-Omega-Hydroxy-L-Arginine	-0.414	4.989	-1.498	0	0	0	3	-0.705
Dodecane-Trimethylamine	-0.410	5.058	-0.857	0	0	6	0	0.190
Hydroxybutyloxide	-0.395	4.175	-1.596	0	0	0	3	0.021
Tris(Hydroxyethyl)Aminomethane	-0.364	5.061	-1.802	0	0	0	0	1.119
Lauroyl chloride	-0.358	4.693	-1.130	0	0	7	0	1.041
12-Hydroxydodecanoic Acid	-0.348	4.696	-0.865	0	0	5	0	0.328
Diethylhomospermine	-0.337	4.687	-1.000	0	0	2	3	-1.132
Oleic Acid	-0.319	5.020	0.232	0	0	12	0	-0.969
Silanol	-0.311	0.000	0.000	0	0	0	0	1.389
N3, N4-Dimethylarginine	-0.295	5.340	-1.269	0	0	0	3	-1.348
Pentaglyme	-0.221	3.939	0.000	0	0	0	0	-1.290
Ocrylate	-0.198	5.679	-0.884	0	0	4	0	-0.429
Isopropyl myristate	-0.188	4.861	-0.134	0	0	10	0	-0.461
Monoctanoin	-0.151	4.802	-1.000	0	0	2	3	-1.211
3,6,9,12,15,18-hexaoxaicosane	-0.112	3.951	0.313	0	0	0	0	-1.848
Vinyl ether	-0.108	4.000	0.000	0	0	0	0	-1.331
NCX 701	-0.094	6.660	0.149	0	0	6	0	-2.670
Trolnitrate	-0.094	5.335	-0.156	1	1	0	0	-1.300
Methyl Nonanoate (Ester)	-0.084	4.780	-1.673	0	0	3	0	1.449
Palmitic Acid	-0.081	4.701	-0.140	0	0	10	0	-0.341
Sebacic acid	-0.070	4.711	-1.291	0	0	3	0	0.824
Azelaic acid	-0.064	4.712	-1.381	0	0	2	0	0.860
Bombykol	-0.004	5.084	-0.189	0	0	10	0	-0.515
Nitrous acid	0.022	2.828	0.000	0	0	0	0	-0.534
L-Homoarginine	0.040	4.989	-1.366	0	0	0	3	-0.938
O-Decyl Hydrogen Thiocarbonate	0.106	4.693	-1.130	0	0	4	0	0.675

Lauric acid	0.148	4.693	-1.130	0	0	6	0	0.919
Spermine	0.161	3.900	-0.868	0	0	2	0	0.508
Quaternium-24	0.164	5.157	0.168	0	0	8	0	-1.437
Rifapentine	0.328	7.907	2.882	1	2	19	3.11	-0.419
Tetraglyme	0.353	3.913	-0.618	0	0	0	0	-0.184
5-(2-hydroxyethyl)nonane-1,9-diol	0.541	4.412	-0.883	0	0	2	0	0.187
Triglyme	0.620	3.864	-1.414	0	0	0	0	1.251
Decane-1-thiol	0.704	3.838	-1.683	0	0	4	0	2.228
Tetrabutylammonium Ion	0.711	5.212	-0.156	0	0	12	0	-0.416
Undecylenic acid	0.719	4.710	-1.366	0	0	5	0	1.200
1-dodecanol	0.953	3.884	-1.136	0	0	6	0	1.480
Bis(hexamethylene)triamine	0.953	3.913	-0.618	0	0	2	0	0.060
N-ethyl-N-[3-(propylamino)propyl]propane-1,3-diamine	1.098	3.900	-0.868	0	0	1	0	0.386
Cetyl alcohol	1.134	3.932	-0.185	0	0	10	0	0.260
Undecanal	1.413	3.959	-1.511	0	0	5	0	1.967
2-{2-[2-2-(Methoxy-Ethoxy)-Ethoxy]-Ethoxy}-Ethanol	1.578	3.900	-0.868	0	0	0	0	0.264
Trolamine salicylate	1.736	6.906	-0.299	1	1	0	0	-2.116
MDL72527	1.843	5.090	-1.037	0	0	4	0	0.240
Nonan-1-Ol	1.980	3.804	-1.902	0	0	3	0	2.516
Hydrogen peroxide	2.196	0.000	0.000	0	0	0	0	1.389
Triethylene glycol	2.428	3.804	-1.902	0	0	0	0	2.150

Table S6. MLRA$_{reg_6LU7}$ external set: compounds identification, 6LU7 Mpro docking score, descriptors value and 6LU7 Mpro docking score predicted.

Compound	Docking score (6LU7)	SpDiam_EA(bo)	Eig09_EA(bo)	nRNR2	N-068	CATS2D_05_LL	nLevel1	Docking score (6LU7) Predicted
**Test active group**								
Coenzyme F420	-10.613	7.581	3.001	0	0	5	3	-9.775
Bleomycin	-10.150	7.161	3.115	0	0	3	3	-9.934
Coa-S-Acetyl 5-Bromotryptamine	-10.141	6.761	2.697	0	0	6	3	-8.561
Carbobenzoxy-Pro-Lys-Phe-Y(Po2)-Ala-Pro-Ome	-10.073	6.644	2.758	0	0	6	3	-8.589
Icatibant	-9.686	7.228	2.709	1	1	9	0	-6.532
BV2	-9.526	7.147	2.961	4	4	4	3	-6.876
Glutathionylspermidine Disulfide	-9.501	7.751	2.868	0	0	45	0	-3.440
Angiotensinamide	-9.137	6.626	2.986	0	0	8	3	-8.734
CBZ-LEU-LEU-TYR-CH2F	-9.039	6.688	2.738	0	0	12	3	-7.852
8-epi-Cyanocobalamin	-8.959	7.687	1.915	0	0	12	0	-5.745
9-hydroxy-6-(3-hydroxypropyl)-4-(2-methoxyphenyl)pyrrolo[3,4-c]carbazole-1,3(2h,6h)-dione	-8.908	7.679	2.082	0	0	7	3	-7.980
Enalkiren	-8.824	7.073	2.481	0	0	3	3	-8.760
Suramin	-8.687	6.756	1.299	0	0	1	3	-6.708
N-{1-[5-(1-Carbamoyl-2-Mercapto-Ethylcarbamoyl)-Pentylcarbamoyl]-2-[4-(Difluoro-Phosphono-Methyl)-Phenyl]-Ethyl}-3-{2-[4-(Difluoro-Phosphono-Methyl)-Phenyl]-Acetylamino}-Succinamic Acid	-8.617	6.626	2.886	0	0	4	3	-9.046
2-(Carboxymethoxy)-5-[(2s)-2-({(2s)-2-[(3-Carboxypropanoyl)Amino] -3-Phenylpropanoyl}Amino)-3-Oxo-3-(Pentylamino)Propyl] Benzoic Acid	-8.552	7.013	2.469	0	0	4	3	-8.575
Delparantag	-8.481	5.319	2.862	0	0	8	3	-7.627
Inhibitor Msa367	-8.216	7.474	3.173	2	2	3	3	-8.923
Fosaprepitant	-8.187	7.581	2.903	0	0	0	3	-10.214
Isavuconazonium	-8.186	7.500	2.754	0	0	8	3	-8.920
Sincalide	-8.181	7.581	2.828	0	0	11	3	-8.739
4-Benzoylamino-4-{1-{1-Carbamoyl-2-[4-	-8.159	6.952	2.183	0	0	0	3	-8.519

(Difluoro-Phosphono-Methyl)-Phenyl]-Ethylcarbamoyl}-2-[4-(Difluoro-Phosphono-Methyl)-Phenyl]-Ethylcarbamoyl}-Butyric Acid								
LFA703	-8.124	7.474	3.014	0	0	8	3	-9.360
Difelikefalin	-8.105	7.025	2.924	0	0	14	3	-8.165
Iomeprol	-8.060	7.500	2.415	0	0	8	3	-8.324
4-(N,N-Dimethylamino)Cinnamoyl-Coa	-8.055	7.557	3.149	0	0	45	3.286	-5.267
Rotigaptide	-8.031	7.581	2.995	0	0	1	3	-10.253
**Test Inactive group**								
Glycine	-1.960	4.435	0	0	0	0	0	-1.627
Aluminiumglycinate	-1.956	4.435	0	0	0	0	0	-1.627
Argininosuccinate	-1.864	5.141	-0.322	0	0	0	3	-2.878
1-Monohexanoyl-2-Hydroxy-Sn-Glycero-3-Phosphate	-1.848	5.717	-1	0	0	0	3	-2.077
(5r)-5-Amino-6-Hydroxyhexylcarbamic Acid	-1.764	4.685	-1.446	0	0	0	3	-0.589
Hypophosphite	-1.739	2.828	0	0	0	0	0	-0.534
Nitroarginine	-1.691	5.404	-1.269	0	0	0	3	-1.391
3-(1-Aminoethyl)Nonanedioic Acid	-1.637	4.891	-1	0	0	3	3	-1.149
N-omega-nitro-L-arginine methyl ester	-1.523	5.404	-0.919	0	0	0	3	-2.006
1-(4-hexylphenyl)prop-2-en-1-one	-1.409	4.776	-0.832	0	0	5	3	-1.122
1-decane-sulfonic-acid	-1.332	6.423	-1.223	0	0	4	0	-0.338
OTX-008	-1.311	7.141	2.824	4	4	32	0	-1.877
Ethambutol	-1.241	4.408	-1	0	0	0	3	-1.187
Linoleic acid	-1.226	5.147	0.238	0	0	12	0	-1.065
Propatyl nitrate	-1.221	5.449	-1.128	0	0	0	0	-0.331
Lauryl Dimethylamine-N-Oxide	-1.185	5.058	-0.857	0	0	6	0	0.19
Stearic acid	-1.174	4.703	0.228	0	0	12	0	-0.747

methoxyundecylphosphinic acid	-1.085	5.759	-0.694	0	0	5	0	-0.696
Decyl(dimethyl)phosphine oxide	-1.053	5.696	-1	0	0	4	0	-0.236
4r-Fluoro-N6-Ethanimidoyl-L-Lysine	-0.926	5.008	-1.268	0	0	0	3	-1.123
S-nonyl-cysteine	-0.898	4.974	-0.703	0	0	4	3	-1.607
4-Oxosebacic Acid	-0.810	4.881	-1.172	0	0	2	0	0.377
Undecyl-Phosphinic Acid Butyl Ester	-0.770	4.811	-0.17	0	0	6	0	-0.851
Octyldodecanol	-0.461	4.447	0.355	0	0	14	3	-1.89
2-octyl cyanoacrylate	-0.429	5.688	-0.787	0	0	5	3	-1.821
11-mercaptoundecanoic acid	-0.318	4.693	-1.13	0	0	4	0	0.675
2-(2-{2-[2-(2-Methoxy-Ethoxy)-Ethoxy]-Ethoxy}-Ethoxy)-Ethanol	-0.253	3.932	-0.185	0	0	0	0	-0.96
Palmidrol	-0.168	4.818	0.314	0	0	10	0	-1.22
undecan-2-one	-0.039	4.686	-1.476	0	0	5	0	1.41
N-Tridecanoic Acid	0.042	4.696	-0.865	0	0	7	0	0.572
Myristic acid	0.072	4.698	-0.601	0	0	8	0	0.229
Capric acid	0.106	4.686	-1.476	0	0	4	0	1.288
Diethylnorspermine	0.113	3.932	-0.185	0	0	0	0	-0.96
N-Octyl-2-Hydroxyethyl Sulfoxide	0.151	4.795	-1.272	0	0	2	0	0.611
Tilarginine	0.199	4.989	-1.498	0	0	0	3	-0.705
1-ethoxy-2-(2-ethoxyethoxy)ethane	0.676	3.838	-1.683	0	0	0	0	1.74
hexadecanal	0.752	3.981	-0.196	0	0	10	0	0.247
decyl formate	0.902	3.966	-1.218	0	0	4	0	1.323
1-(hydroxymethyleneamino)-8-hydroxy-octane	1.075	3.864	-1.414	0	0	1	0	1.373
Trolamine	1.747	4.283	-1.802	1	1	0	0	2.312
Coumermycin A1	2.909	7.299	3.493	0	0	1	3	-10.938

Table S7. ANN_{reg_6LU7} training set: compounds identification, 6LU7 Mpro docking score, descriptors value and 6LU7 Mpro docking score predicted.

Compound	SM4_B(m)	Eig09_EA(bo)	CATS2D_05_LL	s2_relPathLength	Docking score (6LU7)	Docking score (6LU7) Predicted
Gonadorelin	7.954	2.998	6.000	0.046	-11.695	-9.045
Ornipressin	7.773	2.883	0.000	0.022	-11.367	-9.029
Felypressin	7.773	2.710	5.000	0.040	-10.940	-8.947
Carbetocin	7.882	2.153	0.000	0.034	-10.381	-8.857
(E)-(4s,6s)-6-((s)-2-{(s)-2-[(furan-2-carbonyl)-amino]-3-methyl-butyrylamino}-3-methyl-butyrylamino)-8-methyl-5-oxo-4-((r)-2-oxo-pyrrolidin-3-ylmethyl)-non-2-enoic acid ethyl ester	8.146	3.069	3.000	0.042	-10.222	-9.039
Iotrolan	10.600	1.360	0.000	0.000	-10.176	-8.843
Lypressin	7.244	2.376	28.000	0.037	-10.107	-3.920
Triptorelin	8.089	3.139	9.000	0.043	-10.045	-9.192
Lanreotide	7.312	2.077	3.000	0.057	-9.973	-8.686
Birinapant	7.585	2.705	16.000	0.022	-9.893	-5.768
Indium In-111 pentetreotide	8.025	3.050	8.000	0.031	-9.877	-9.248
Ipamorelin	10.602	2.099	4.000	0.000	-9.873	-9.593
UK-432097	7.580	2.676	12.000	0.026	-9.697	-7.783
Pentagastrin	7.381	2.093	1.000	0.038	-9.648	-8.767
Terlipressin	8.591	3.971	28.000	0.000	-9.511	-5.737
Succinamide-Coa	8.071	3.012	6.000	0.046	-9.472	-9.063
Iopamidol	11.292	2.393	0.000	0.000	-9.397	-9.754
BV1	7.523	2.842	3.000	0.026	-9.387	-9.034
Labradimil	7.608	2.815	8.000	0.026	-9.377	-8.976
Etelcalcetide	7.250	2.506	5.000	0.060	-9.353	-8.781
LY231514 Tetra Glu	7.356	2.642	5.000	0.051	-9.318	-8.846
talactoferrin alpha	7.848	2.911	1.000	0.044	-9.217	-8.955
8-Demethyl-8-Dimethylamino-Flavin-Adenine-Dinucleotide	6.817	1.200	8.000	0.136	-9.206	-7.617
N-(Sulfanylacetyl)Tyrosylprolylmethioninamide	6.667	0.953	3.000	0.080	-9.162	-7.253
Colistin	7.626	2.775	3.000	0.013	-9.125	-9.129
4'-nitrophenyl-3i-thiolaminaritrioside	7.798	2.914	0.000	0.016	-9.014	-9.079
Hydroxyethyl cellulose	7.954	2.998	6.000	0.046	-9.014	-9.045
S-(2-Oxo)Pentadecylcoa	7.560	2.683	5.000	0.069	-9.000	-8.854
4-Hydroxyphenacyl Coenzyme A	7.967	3.002	4.000	0.039	-8.999	-9.052
Coa-S-Acetyl Tryptamine	8.557	3.006	5.000	0.038	-8.927	-9.170
Vapreotide	7.987	3.142	5.000	0.059	-8.904	-8.984
Adrabetadex	7.836	2.571	0.000	0.088	-8.894	-8.862

Thymopentin	7.349	2.654	1.000	0.042	-8.889	-8.906
Saquinavir	7.815	2.941	9.000	0.041	-8.844	-9.015
3-Thiaoctanoyl-Coenzyme A	6.932	1.310	5.000	0.100	-8.745	-7.986
4-methyl-pentanoic acid {1-[4-guanidino-1-(thiazole-2-carbonyl)-butylcarbamoyl]-2-methyl-propyl}-amide	7.806	2.743	0.000	0.045	-8.745	-8.923
Sinapoyl Coenzyme A	7.276	2.629	4.000	0.036	-8.736	-8.884
P1-(5'-Adenosyl)P5-(5'-Thymidyl)Pentaphosphate	7.093	1.500	3.000	0.083	-8.726	-8.429
Dotatate	7.547	2.890	16.000	0.080	-8.708	-7.711
3-[3-(2,3-Dihydroxy-Propylamino)-Phenyl]-4-(5-Fluoro-1-Methyl-1h-Indol-3-Yl)-Pyrrole-2,5-Dione	7.307	2.140	0.000	0.050	-8.697	-8.817
Iobitridol	7.779	2.803	5.000	0.086	-8.697	-8.867
Angiotensin II	7.800	2.989	8.000	0.037	-8.674	-9.095
Flavin adenine dinucleotide	7.838	2.880	4.000	0.064	-8.539	-8.915
Iodixanol	7.509	2.484	0.000	0.048	-8.538	-8.881
Sufugolix	6.757	1.493	1.000	0.071	-8.501	-8.536
Elamipretide	7.802	2.877	19.000	0.034	-8.476	-5.487
Flavin-N7 Protonated-Adenine Dinucleotide	7.827	2.862	4.000	0.015	-8.446	-9.203
Alatrofloxacin	7.305	2.461	10.000	0.025	-8.428	-7.575
Methylmalonyl-Coenzyme A	7.605	2.749	14.000	0.034	-8.428	-7.304
Coa-S-Trimethylene-Acetyl-Tryptamine	7.999	2.994	4.000	0.038	-8.422	-9.063
Trifluoroacetonyl Coenzyme A	7.996	2.926	4.000	0.068	-8.388	-8.919
Je-2147, Ag1776, Kni-764	7.472	2.784	9.000	0.087	-8.329	-8.771
Giripladib	7.095	2.046	1.000	0.061	-8.314	-8.768
Efrotomycin	7.248	2.120	9.000	0.058	-8.288	-7.537
3-[1-(3-aminopropyl)-1h-indol-3-yl]-4-(1h-indol-3-yl)-1h-pyrrole-2,5-dione	7.922	2.825	4.000	0.042	-8.259	-8.992
Fosifloxuridine nafalbenamide	7.436	2.322	3.000	0.065	-8.250	-8.802
HM-30181	7.515	1.904	2.000	0.060	-8.238	-8.691
2-Hydroxy-5-({1-[(4-Methylphenoxy)Methyl]-3-Oxoprop-1-Enyl}Amino)-L-Tyrosine	6.876	1.456	4.000	0.000	-8.232	-5.260
Caspofungin	7.398	2.322	5.000	0.065	-8.208	-8.719
Benzoyl-Arginine-Alanine-Methyl Ketone	6.749	1.348	1.000	0.107	-8.200	-8.670
Inhibitor Bea388	7.568	2.324	0.000	0.049	-8.191	-8.861
3-[(5s)-1-Acetyl-3-(2-Chlorophenyl)-4,5-Dihydro-1h-Pyrazol-5-Yl]Phenol	7.935	3.001	4.000	0.041	-8.186	-9.036
Cefbuperazone	7.148	2.153	2.000	0.075	-8.171	-8.788
Telinavir	7.555	2.597	10.000	0.000	-8.164	-8.023
Nicotinamide-Adenine-Dinucleotide-5-Hydroxy-4-Oxonorvaline	7.773	2.883	0.000	0.022	-8.154	-9.029

Cangrelor	7.878	2.961	4.000	0.014	-8.151	-9.258
Polymyxin B	7.846	2.886	4.000	0.066	-8.149	-8.912
N-(4-carbamimidoylbenzyl)-1-(4-methylpentanoyl)-L-prolinamide	7.892	2.911	2.000	0.036	-8.129	-9.006
NUC-1031	7.817	2.807	3.000	0.076	-8.118	-8.883
Larazotide	7.845	2.901	5.000	0.032	-8.117	-9.085
Inarigivir soproxil	7.250	2.210	0.000	0.048	-8.105	-8.829
Cephalosporin Analog	7.431	2.110	0.000	0.113	-8.077	-8.840
FR236913	7.470	2.427	3.000	0.029	-8.056	-8.857
TOP-1288	7.292	2.427	3.000	0.061	-8.054	-8.820
Ioforminol	10.602	1.689	4.000	0.000	-8.050	-8.901
Dirlotapide	7.326	2.096	5.000	0.066	-8.034	-8.574
BMS-986094	8.170	3.115	3.000	0.021	-8.021	-9.224
Iopentol	10.600	1.239	0.000	0.000	-8.016	-8.630
Carfilzomib	7.536	2.160	5.000	0.054	-8.009	-8.582
4-Oxo-Nicotinamide-Adenine Dinucleotide Phosphate	7.110	2.085	15.000	0.000	-8.002	-3.431
Agmatine	5.379	0.000	0.000	0.000	-2.000	-0.999
1-acetyl-2-lyso-sn-glycero-3-phosphoethanolamine	6.537	-1.000	0.000	0.000	-1.961	-0.316
Glycerin	4.893	0.000	0.000	0.000	-1.961	-0.901
Magnesium glycinate	5.731	0.000	0.000	0.000	-1.960	-1.078
Zinc glycinate	7.065	0.000	0.000	0.000	-1.960	-1.446
Aluminum zirconium octachlorohydrex gly	8.184	0.000	0.000	0.000	-1.956	-1.862
Dihydroxyacetone	5.121	0.000	0.000	0.000	-1.956	-0.946
Ferrous glycine sulfate	7.147	-1.372	0.000	0.000	-1.956	-0.263
(S)-2-Amino-4-[(2s,3r)-2,3,5-Trihydroxy-4-Oxo-Pentyl]Mercapto-Butyric Acid	6.376	-0.750	0.000	0.000	-1.908	-0.401
1,4-Butanediol	4.760	0.000	0.000	0.000	-1.859	-0.876
Amyl Nitrite	5.281	0.000	0.000	0.000	-1.822	-0.979
3-Oxo-Pentadecanoic Acid	6.089	-0.355	8.000	0.000	-1.815	-0.487
4-Hydroxybutan-1-Aminium	4.724	0.000	0.000	0.000	-1.809	-0.870
NB-001	6.424	0.421	0.000	0.000	-1.783	-2.578
L-cystein-s-1-(iminomethyl)-l-ornithine	6.457	-0.774	1.000	0.000	-1.733	-0.355
L-Alpha-Glycerophosphorylserine	6.556	-1.000	0.000	0.000	-1.729	-0.317
2-Hydroxyethyl Disulfide	5.968	0.000	0.000	0.000	-1.698	-1.135
Ricinoleic Acid	6.195	0.232	10.000	0.000	-1.612	-0.772
cis-Vaccenic acid	6.119	0.235	12.000	0.000	-1.610	-0.950
Tylosin	7.507	2.468	16.000	0.039	-1.587	-4.618
Dexpanthenol	5.902	-1.334	1.000	0.000	-1.578	-0.212
Nz-(1-Carboxyethyl)-Lysine	6.041	-1.262	1.000	0.000	-1.566	-0.226
Troleandomycin	7.445	2.601	17.000	0.035	-1.566	-4.688

N-(Phosphonoacetyl)-L-Ornithine	6.590	-1.000	0.000	0.000	-1.542	-0.319
Sevelamer	5.871	0.000	0.000	0.000	-1.522	-1.112
3,7,11,15-tetramethyl-hexadecan-1-ol	6.069	-0.090	16.000	0.000	-1.515	-1.440
Nz-(Dicarboxymethyl)Lysine	6.249	-1.095	0.000	0.000	-1.508	-0.274
L-Alpha-Glycerophosphorylethanolamine	6.358	-1.365	0.000	0.000	-1.502	-0.229
1,3-Propandiol	4.582	0.000	0.000	0.000	-1.482	-0.844
N-Alpha-L-Acetyl-Arginine	6.118	-1.316	1.000	0.133	-1.475	-1.279
Methylethylamine	4.236	0.000	0.000	0.000	-1.437	-0.786
(9Z,11E,13S)-13-hydroxyoctadeca-9,11-dienoic acid	6.246	0.420	10.000	0.000	-1.410	-0.851
(3r)-3-hydroxydodecanoic acid	6.188	-0.502	10.000	0.000	-1.409	-0.604
N-Butyl-N'-Hydroxyguanidine	5.400	0.000	0.000	0.000	-1.371	-1.004
2-Decenoyl N-Acetyl Cysteamine	6.358	-0.267	6.000	0.000	-1.357	-0.466
4-amino-n-[(2-sulfanylethyl)carbamoyl]benzenesulfonamide	6.881	-0.602	0.000	0.000	-1.335	-0.539
Cimetidine	6.497	-0.206	0.000	0.000	-1.306	-0.901
Undecylamine-n,n-dimethyl-n-oxide	5.761	-1.000	5.000	0.000	-1.302	-0.274
MF268	6.109	-0.128	3.000	0.000	-1.288	-0.541
Triethoxycaprylylsilane	6.237	-0.445	5.000	0.000	-1.283	-0.392
Ethyl Isocyanide	4.849	0.000	0.000	0.000	-1.203	-0.893
Alpha-Linolenic Acid	6.227	0.251	12.000	0.000	-1.198	-0.985
Sodium lauryl sulfate	6.616	-0.594	6.000	0.000	-1.178	-0.426
Dodecyl sulfate	6.597	-0.594	6.000	0.000	-1.174	-0.424
Magnesium stearate	6.771	1.477	24.000	0.000	-1.174	-3.949
Guanidine-3-Propanol	5.307	0.000	0.000	0.000	-1.153	-0.984
Dolastatin 10	7.487	2.239	10.000	0.032	-1.137	-6.920
(10E,12Z)-octadecadienoic acid	6.175	0.242	12.000	0.000	-1.114	-0.968
S-(D-Carboxybutyl)-L-Homocysteine	6.250	-1.239	1.000	0.000	-1.108	-0.238
N2-(Carboxyethyl)-L-Arginine	6.213	-0.896	1.000	0.118	-1.104	-1.995
Diethyl propane-1,3-diylbiscarbamate	6.008	-0.737	0.000	0.000	-1.072	-0.378
GW-274150	6.195	-1.275	1.000	0.000	-1.060	-0.231
Etoglucid	6.200	0.000	0.000	0.000	-1.057	-1.194
Palmitoleic Acid	6.031	-0.147	10.000	0.000	-1.019	-0.643
Odalasvir	7.843	2.999	54.000	0.041	-1.013	-4.910
3-Hydroxy-Myristic Acid	5.961	-0.492	7.000	0.000	-1.002	-0.406
Nitrosoethane	4.693	0.000	0.000	0.000	-0.974	-0.864
Elaidoylamide	6.110	0.232	12.000	0.000	-0.953	-0.947
Bromo-Dodecanol	7.806	-0.868	6.000	0.000	-0.947	-0.530
Ethylhexylglycerin	5.695	-1.128	2.000	0.071	-0.914	-0.357

Rifampicin	7.580	2.718	19.000	0.019	-0.888	-4.668
1-Guanidinium-7-Aminoheptane	5.486	-1.328	0.000	0.000	-0.881	-0.204
Ethanolamine oleate	6.269	0.602	12.000	0.000	-0.866	-1.124
Ethanol	3.957	0.000	0.000	0.000	-0.776	-0.742
C31G	6.659	1.428	12.000	0.000	-0.753	-1.933
N-dodecyl-n,n-dimethylglycinate	6.095	-0.348	6.000	0.000	-0.753	-0.417
5-N-Allyl-Arginine	6.055	-1.259	0.000	0.000	-0.752	-0.234
Hexaminolevulinate	5.963	-1.037	2.000	0.000	-0.718	-0.251
Cetrimonium	6.004	0.070	10.000	0.000	-0.607	-0.687
Dibutylsuccinate	6.030	-0.482	4.000	0.000	-0.599	-0.359
2-[3-(2-Hydroxy-1,1-Dihydroxymethyl-Ethylamino)-Propylamino]-2-Hydroxymethyl-Propane-1,3-Diol	6.100	0.213	0.000	0.000	-0.567	-1.690
Tiadenol	6.345	0.000	5.000	0.000	-0.524	-0.560
N5-(1-Imino-3-Butenyl)-L-Ornithine	5.929	-1.268	2.000	0.000	-0.514	-0.222
3,6,9,12,15-Pentaoxaheptadecane	5.903	-0.185	0.000	0.000	-0.513	-0.820
N-Omega-Propyl-L-Arginine	5.992	-1.143	0.000	0.000	-0.511	-0.252
Dioctyldimonium	5.949	0.072	6.000	0.000	-0.501	-0.526
Decamethonium	6.001	-0.845	3.000	0.000	-0.459	-0.283
11-[(mercaptocarbonyl)oxy]undecanoic acid	6.259	-0.582	4.000	0.000	-0.453	-0.355
N-Omega-Hydroxy-L-Arginine	5.914	-1.498	0.000	0.000	-0.414	-0.198
Dodecane-Trimethylamine	5.796	-0.857	6.000	0.000	-0.410	-0.314
Hydroxybutyloxide	5.494	-1.596	0.000	0.000	-0.395	-0.178
Tris(Hydroxyethyl)Aminomethane	5.502	-1.802	0.000	0.000	-0.364	-0.165
Lauroyl chloride	6.060	-1.130	7.000	0.000	-0.358	-0.342
12-Hydroxydodecanoic Acid	5.831	-0.865	5.000	0.000	-0.348	-0.292
Diethylhomospermine	5.691	-1.000	2.000	0.000	-0.337	-0.244
Oleic Acid	6.119	0.232	12.000	0.000	-0.319	-0.949
Silanol	4.468	0.000	0.000	0.000	-0.311	-0.825
N3, N4-Dimethylarginine	5.951	-1.269	0.000	0.000	-0.295	-0.228
Pentaglyme	5.987	0.000	0.000	0.000	-0.221	-1.140
Ocrylate	6.076	-0.884	4.000	0.000	-0.198	-0.290
Isopropyl myristate	6.043	-0.134	10.000	0.000	-0.188	-0.647
Monoctanoin	5.883	-1.000	2.000	0.000	-0.151	-0.253
3,6,9,12,15,18-hexaoxaicosane	6.074	0.313	0.000	0.000	-0.112	-2.000
Vinyl ether	4.967	0.000	0.000	0.000	-0.108	-0.916
NCX 701	6.595	0.149	6.000	0.000	-0.094	-0.665
Trolnitrate	6.605	-0.156	0.000	0.000	-0.094	-1.002
Methyl Nonanoate (Ester)	5.630	-1.673	3.000	0.000	-0.084	-0.193

Palmitic Acid	5.967	-0.140	10.000	0.000	-0.081	-0.632
Sebacic acid	5.925	-1.291	3.000	0.000	-0.070	-0.228
Azelaic acid	5.873	-1.381	2.000	0.000	-0.064	-0.210
Bombykol	5.915	-0.189	10.000	0.000	-0.004	-0.613
Nitrous acid	4.600	0.000	0.000	0.000	0.022	-0.848
L-Homoarginine	5.892	-1.366	0.000	0.000	0.040	-0.212
O-Decyl Hydrogen Thiocarbonate	6.005	-1.130	4.000	0.000	0.106	-0.259
Lauric acid	5.750	-1.130	6.000	0.000	0.148	-0.286
Spermine	5.602	-0.868	2.000	0.000	0.161	-0.261
Quaternium-24	6.210	0.168	8.000	0.000	0.164	-0.637
Rifapentine	7.628	2.882	19.000	0.018	0.328	-5.312
Tetraglyme	5.799	-0.618	0.000	0.000	0.353	-0.421
3,6,9,12,15-pentaoxaheptadecan-1-ol	5.973	0.000	0.000	0.000	-0.471	-1.136
5-(2-hydroxyethyl)nonane-1,9-diol	5.652	-0.883	2.000	0.000	0.541	-0.261
Triglyme	5.568	-1.414	0.000	0.000	0.620	-0.196
Decane-1-thiol	5.651	-1.683	4.000	0.000	0.704	-0.207
Tetrabutylammonium Ion	5.832	-0.156	12.000	0.000	0.711	-0.780
Undecylenic acid	5.768	-1.366	5.000	0.000	0.719	-0.247
1-dodecanol	5.491	-1.136	6.000	0.000	0.953	-0.269
Bis(hexamethylene)triamine	5.652	-0.618	2.000	0.000	0.953	-0.322
N-ethyl-N-[3-(propylamino)propyl]propane-1,3-diamine	5.595	-0.868	1.000	0.000	1.098	-0.275
Cetyl alcohol	5.764	-0.185	10.000	0.000	1.134	-0.588
Undecanal	5.534	-1.511	5.000	0.000	1.413	-0.228
2-{2-[2-2-(Methoxy-Ethoxy)-Ethoxy]-Ethoxy}-Ethanol	5.725	-0.868	0.000	0.000	1.578	-0.310
Trolamine salicylate	6.366	-0.299	0.000	0.000	1.736	-0.754
MDL72527	5.931	-1.037	4.000	0.000	1.843	-0.263
Nonan-1-Ol	5.222	-1.902	3.000	0.000	1.980	-0.173
Hydrogen peroxide	3.583	0.000	0.000	0.000	2.196	-0.688
Triethylene glycol	5.370	-1.902	0.000	0.000	2.428	-0.157

Table S8. ANN_{reg_6LU7} external set: compounds identification, 6LU7 Mpro docking score, descriptors value and 6LU7 Mpro docking score predicted.

Compound	SM4_B(m)	Eig09_EA(bo)	CATS2D_05_LL	s2_relPathLength	Docking score (6LU7)	Docking score (6LU7) Predicted
**Test active group**						
Coenzyme F420	8.018	3.001	5	0.036	-10.613	-9.104
Bleomycin	8.170	3.115	3	0.021	-10.150	-9.224
Coa-S-Acetyl 5-Bromotryptamine	7.514	2.697	6	0.064	-10.141	-8.841
Carbobenzoxy-Pro-Lys-Phe-Y(Po2)-Ala-Pro-Ome	7.730	2.758	6	0.067	-10.073	-8.872
Icatibant	7.533	2.709	9	0.000	-9.686	-8.720
BV2	7.866	2.961	4	0.015	-9.526	-9.248
Glutathionylspermidine Disulfide	7.759	2.868	45	0.000	-9.501	-4.907
Angiotensinamide	7.790	2.986	8	0.041	-9.137	-9.063
CBZ-LEU-LEU-TYR-CH2F	7.714	2.738	12	0.030	-9.039	-8.141
8-epi-Cyanocobalamin	7.052	1.915	12	0.000	-8.959	-3.301
9-hydroxy-6-(3-hydroxypropyl)-4-(2-methoxyphenyl)pyrrolo [3,4-c]carbazole-1,3(2h,6h)-dione	7.037	2.082	7	0.000	-8.908	-6.702
Enalkiren	7.276	2.481	3	0.071	-8.824	-8.832
Suramin	6.735	1.299	1	0.074	-8.687	-8.393
N-{1-[5-(1-Carbamoyl-2-Mercapto-Ethylcarbamoyl)-Pentylcarbamoyl]-2-[4-(Difluoro-Phosphono-Methyl)-Phenyl]-Ethyl}-3-{2-[4-(Difluoro-Phosphono-Methyl)-Phenyl]-Acetylamino}-Succinamic Acid	7.854	2.886	4	0.067	-8.617	-8.910
2-(Carboxymethoxy)-5-[(2s)-2-({(2s)-2-[(3-Carboxypropanoyl)Amino] -3-Phenylpropanoyl}Amino)-3-Oxo-3-(Pentylamino)Propyl]Benzoic Acid	7.256	2.469	4	0.140	-8.552	-8.840
Delparantag	7.750	2.862	8	0.037	-8.481	-8.991
Inhibitor Msa367	9.364	3.173	3	0.043	-8.216	-9.167
Fosaprepitant	7.775	2.903	0	0.016	-8.187	-9.071
Isavuconazonium	11.295	2.754	8	0.015	-8.186	-10.182
Sincalide	7.930	2.828	11	0.038	-8.181	-8.746
4-Benzoylamino-4-{1-{1-Carbamoyl-2-[4-	7.281	2.183	0	0.040	-8.159	-8.824

(Difluoro-Phosphono-Methyl)-Phenyl]-Ethylcarbamoyl}-2-[4-(Difluoro-Phosphono-Methyl)-Phenyl]-Ethylcarbamoyl}-Butyric Acid						
LFA703	7.942	3.014	8	0.052	-8.124	-9.023
Difelikefalin	7.864	2.924	14	0.046	-8.105	-8.294
Iomeprol	11.293	2.415	8	0.000	-8.060	-10.033
4-(N,N-Dimethylamino)Cinnamoyl-Coa	8.265	3.149	45	0.044	-8.055	-4.880
Rotigaptide	8.047	2.995	1	0.029	-8.031	-9.053
**Test inactive group**						
Glycine	4.985	0.000	0	0.000	-1.960	-0.919
Aluminiumglycinate	5.199	0.000	0	0.000	-1.956	-0.962
Argininosuccinate	6.436	-0.322	0	0.050	-1.864	-1.862
1-Monohexanoyl-2-Hydroxy-Sn-Glycero-3-Phosphate	6.565	-1.000	0	0.000	-1.848	-0.318
(5r)-5-Amino-6-Hydroxyhexylcarbamic Acid	5.708	-1.446	0	0.000	-1.764	-0.197
Hypophosphite	5.490	0.000	0	0.000	-1.739	-1.024
Nitroarginine	6.242	-1.269	0	0.000	-1.691	-0.240
3-(1-Aminoethyl)Nonanedioic Acid	6.074	-1.000	3	0.063	-1.637	-0.319
N-omega-nitro-L-arginine methyl ester	6.283	-0.919	0	0.000	-1.523	-0.326
1-(4-hexylphenyl)prop-2-en-1-one	5.858	-0.832	5	0.000	-1.409	-0.298
1-decane-sulfonic-acid	6.496	-1.223	4	0.000	-1.332	-0.279
OTX-008	7.679	2.824	32	0.000	-1.311	-4.812
Ethambutol	5.690	-1.000	0	0.143	-1.241	-3.271
Linoleic acid	6.175	0.238	12	0.000	-1.226	-0.967
Propatyl nitrate	6.586	-1.128	0	0.000	-1.221	-0.284
Lauryl Dimethylamine-N-Oxide	5.819	-0.857	6	0.000	-1.185	-0.316
Stearic acid	6.060	0.228	12	0.000	-1.174	-0.932
methoxyundecylphosphinic acid	6.388	-0.694	5	0.000	-1.085	-0.358

Decyl(dimethyl)phosphine oxide	6.294	-1.000	4	0.000	-1.053	-0.289
4r-Fluoro-N6-Ethanimidoyl-L-Lysine	5.997	-1.268	0	0.000	-0.926	-0.230
S-nonyl-cysteine	6.168	-0.703	4	0.000	-0.898	-0.324
4-Oxosebacic Acid	6.096	-1.172	2	0.000	-0.810	-0.240
Undecyl-Phosphinic Acid Butyl Ester	6.339	-0.170	6	0.000	-0.770	-0.491
Octyldodecanol	5.999	0.355	14	0.095	-0.461	-0.349
2-octyl cyanoacrylate	6.097	-0.787	5	0.000	-0.429	-0.321
11-mercaptoundecanoic acid	5.996	-1.130	4	0.000	-0.318	-0.258
2-(2-{2-[2-(2-Methoxy-Ethoxy)-Ethoxy]-Ethoxy}-Ethoxy)-Ethanol	5.926	-0.185	0	0.000	-0.253	-0.824
Palmidrol	6.110	0.314	10	0.000	-0.168	-0.780
undecan-2-one	5.593	-1.476	5	0.000	-0.039	-0.232
N-Tridecanoic Acid	5.809	-0.865	7	0.000	0.042	-0.345
Myristic acid	5.864	-0.601	8	0.000	0.072	-0.422
Capric acid	5.621	-1.476	4	0.000	0.106	-0.216
Diethylnorspermine	5.803	-0.185	0	0.000	0.113	-0.803
N-Octyl-2-Hydroxyethyl Sulfoxide	6.178	-1.272	2	0.000	0.151	-0.232
Tilarginine	5.893	-1.498	0	0.000	0.199	-0.197
1-ethoxy-2-(2-ethoxyethoxy)ethane	5.438	-1.683	0	0.000	0.676	-0.170
hexadecanal	5.853	-0.196	10	0.000	0.752	-0.601
decyl formate	5.646	-1.218	4	0.000	0.902	-0.233
1-(hydroxymethyleneamino)-8-hydroxy-octane	5.464	-1.414	1	0.000	1.075	-0.191
Trolamine	5.375	-1.802	0	0.000	1.747	-0.162
Coumermycin A1	7.969	3.493	1	0.031	2.909	-9.079

Table S9. Descriptors' value for the selected anti-SARS-coV-2 compounds.

Compound	MPC08	SpDiam_ EA(bo)	Eig09_ EA(bo)	nRNR2	N-068	CATS2D _05_LL	nLevel1	SM4_B(m)	s2_relPath Length
Docetaxol	6.232	7.196	2.654	0	0	15	3.273	7.576	0.056
Ginsenoside	6.489	6.391	2.638	0	0	32	3.154	7.519	0.040
Josamycin	5.493	6.035	2.400	1	1	13	3.063	7.420	0.058
[(3R,6S)-3,4,5-tris(acetyloxy)-6-{4-[bis(2-hydroxyethyl)carbamoyl]-2-methoxyphenoxy}oxan-2-yl]methyl acetate (Molport-046-067-769)	5.193	6.985	2.261	0	0	0	3.000	7.189	0.093
(2S,5S)-2-[(4-methoxyphenyl)methyl]-4,5-dimethyl-11-[4-oxo-4-(2,4,5-trimethoxyphenyl)butanoyl]-1,4,7,11-tetraazacyclopentadecane-3,6,15-trione (Molport-046-568-802)	4.836	7.137	2.559	0	0	9	3.000	7.327	0.074
Pepstatin A	4.220	5.307	2.100	0	0	10	3.000	7.221	0.030
